# Joint Dietary and Gut Microbial Profiling and the Fatty Liver Index in Community-Dwelling Older Japanese: A Cross-Sectional, Hypothesis-Generating Analysis from the Kyotango Longevity Study

**DOI:** 10.3390/nu18142300

**Published:** 2026-07-14

**Authors:** Yuji Naito, Takeshi Yasuda, Hiroaki Kitae, Katsura Mizushima, Norihiro Ouchi, Atsuo Adachi, Tadaaki Kamitani, Jin Narumoto, Tomoya Kitani, Satoaki Matoba, Ryo Inoue, Tomohisa Takagi

**Affiliations:** 1 Department of Human Immunology and Nutrition Science, Graduate School of Medical Science, Kyoto Prefectural University of Medicine, Kyoto 602-8566, Japan; 2 Molecular Gastroenterology and Hepatology, Graduate School of Medical Science, Kyoto Prefectural University of Medicine, Kyoto 602-8566, Japan; 3 Department of Internal Medicine, Kyotango Municipal Yasaka Hospital, Kyoto 627-0111, Japan; 4 Department of Psychiatry, Graduate School of Medical Science, Kyoto Prefectural University of Medicine, Kyoto 602-8566, Japan; 5 Department of Cardiovascular Medicine, Graduate School of Medical Science, Kyoto Prefectural University of Medicine, Kyoto 602-8566, Japan; 6 Department of Longevity and Regional Epidemiology, Graduate School of Medical Science, Kyoto Prefectural University of Medicine, Kyoto 602-8566, Japan; 7 Laboratory of Animal Science, Department of Applied Biological Sciences, Faculty of Agriculture, Setsunan University, Osaka 573-0101, Japan

**Keywords:** dietary pattern, gut microbiota, fatty liver index (FLI), canonical correlation analysis, mediation analysis, hypothesis-generating, older adults, community-dwelling cohort, Kyotango

## Abstract

Background: Diet and the gut microbiota are each associated with hepatic steatosis, but their joint variation and shared explanatory contribution are rarely quantified in older Asian community-dwelling populations. Methods: In 701 non-heavy-drinking Kyotango longevity cohort adults, habitual diet (BDHQ; 31 food groups) and stool 16S rRNA microbiome (47 genera; CLR-transformed) were related to the fatty liver index (FLI) by canonical correlation analysis (CCA), reduced-rank regression (RRR), and bootstrap mediation; FIB-4 was a secondary exploratory outcome. Results: Four dietary patterns and four microbial clusters emerged. CCA revealed multivariate diet-microbiota co-variation (four significant canonical correlations; r = 0.40–0.46; all *p* < 0.05). Combined RRR (*n* = 697 with complete FLI data) explained 11.1% of FLI variance in-sample (permutation *p* = 0.006), although cross-validated R^2^ was negative, reframing the model as hypothesis-generating rather than predictive. Bootstrap mediation suggested that 12.6% of the diet-on-FLI effect was carried by the microbiota (95% bootstrap CI excluding zero). Of 1457 FDR-corrected food-genus pairs, one was significant (fruits × *Eubacterium eligens*; r = +0.202, q = 1.0 × 10^−4^). Conclusions: In this cross-sectional, hypothesis-generating analysis with no individual-level predictive utility, dietary patterns and gut microbial composition co-vary and jointly relate to FLI. The findings describe population-level covariance patterns for future prospective diet-microbiome intervention testing; external validation in independent cohorts is essential.

## 1. Introduction

Non-alcoholic fatty liver disease (NAFLD), recently reframed as metabolic dysfunction–associated steatotic liver disease (MASLD) under the 2023 multi-society Delphi consensus on fatty liver disease nomenclature [1] and the 2024 EASL–EASD–EASO clinical practice guidelines [2], is currently the leading chronic liver condition worldwide, with a global prevalence of ~30% of adults that continues to rise parallel with the obesity and type 2 diabetes pandemics [2,3,4]. In Japan, MASLD prevalence has been increasing with population aging and the gradual westernization of dietary habits [5]. This has prompted the Japan Society of Gastroenterology and Hepatology to issue the 2026 evidence-based clinical practice guidelines for MASLD, which provide Japan-specific recommendations on diagnosis, risk stratification, and management aligned with the international MASLD/MetALD framework [6]. Because no metabolic dysfunction-associated steatohepatitis (MASH)-targeted pharmacotherapy can currently be recommended for a non-cirrhotic disease beyond the conditional use of resmetirom in selected patients [2], lifestyle modification—weight loss, dietary changes, physical exercise, and discouraging alcohol consumption—remains the cornerstone of management for the majority of patients [2]. Against this background, the emerging framework of preventive hepatology—reframing care from late-stage treatment toward early, population-level identification, and modification of upstream risk factors—has become the most actionable strategy to curb the rising MASLD burden, particularly in regions undergoing rapid metabolic transition [4,7]. Validated non-invasive surrogates support such population-scale approaches. The fatty liver index (FLI) integrates body-mass index (BMI), waist circumference, serum triglycerides, and γ-glutamyltransferase, and provides a reliable continuous estimate of hepatic steatosis [8]. The Fibrosis-4 (FIB-4) index combines age, aspartate aminotransferase (AST), alanine aminotransferase (ALT), and platelet count to estimate advanced fibrosis risk [9] and is recommended as the first-tier non-invasive test in the EASL stepwise pathway for ruling out advanced fibrosis [2]. Together, FLI and FIB-4 enable population-scale characterization of two distinct yet interconnected liver phenotypes—steatosis and fibrosis.

Diet and gut microbiome are two of the most accessible modifiable factors influencing liver health [10,11,12]. The gut–liver axis hypothesis posits that gut-derived microbial metabolites and bacterial translocation across the intestinal barrier modulate hepatocyte metabolism and Kupffer cell activation, contributing to steatosis, inflammation, and fibrogenesis [12,13]. Diet directly shapes microbial composition, particularly through fiber availability for saccharolytic fermenters [14] and fat- and protein-rich substrates favoring proteolytic and bile-acid-modifying bacteria [15]. Specific dietary components have been mechanistically implicated in microbiome-mediated MASLD progression. In mice, both dietary cholesterol and saturated fat are required to drive fibrosing MASH; their combination reshapes the gut microbiota and bile-acid pool such that diluted cecal contents from high-fat, high-cholesterol-fed specific-pathogen-free (but not germ-free) mice activate human hepatic stellate cells in vitro, providing direct evidence of a Western-diet-induced microbiota–fibrogenesis axis [16]. Alcohol intake adds another layer to this crosstalk: even moderate alcohol consumption combined with metabolic risk factors can exacerbate, compounds gut barrier dysfunction and intestinal dysbiosis, thereby accelerating the progression from steatosis to steatohepatitis and fibrosis under the recently introduced MetALD framework [17]. A logical implication is that diet and the gut microbiome should be considered jointly, rather than as independent exposures, when evaluating liver phenotypes. However, most epidemiological studies analyze each modality separately, making it unresolved whether the two contributions are redundant, complementary, or partially statistically accounted for.

This is particularly relevant in older adults. Aging is accompanied by changes in dietary intake (decreased appetite; altered taste; social isolation); shifts in microbiome composition (decreased *Bifidobacterium*; increased *Clostridium difficile* risk; *Christensenellaceae* enrichment in healthy centenarians [18]), and liver phenotype changes that confound surrogate indices. FIB-4 explicitly includes age in its formula; consequently increasing with age regardless of true fibrosis [19], which complicates its use in elderly cohorts.

In this study, we utilized the data from 701 community-dwelling residents (age 65–101 years) of the Kyotango region—a Kyotango longevity area in northern Kyoto Prefecture, Japan, characterized by one of the highest centenarian densities in the country and previously profiled for its distinctive gut microbiota composition compared with the neighboring urban populations [20,21]—to test three hypotheses. (i) Dietary patterns and gut microbial composition co-vary in a multivariate, descriptive sense. (ii) Integrated modeling of diet and microbiome explains a larger fraction of variance in hepatic steatosis (FLI [8]) compared with either modality alone, with FIB-4 [9,22] examined as a secondary exploratory outcome. (iii) The gut microbiome statistically accounts for part of the observed diet-FLI association on hepatic phenotypes. We addressed the FIB-4 age-confounding problem with age-residualization and avoided over-adjustment by excluding variables embedded in the FLI/FIB-4 formulae. To our knowledge, this is the first study to perform a multi-response reduced-rank regression (RRR) modeling diet + microbiome together against FLI and FIB-4 in an elderly Asian cohort, complemented by canonical correlation analysis and bootstrap mediation. By framing the diet-microbiome-FLI triad as a partially statistically accounted for joint association rather than as separate independent exposures, our findings can support the preventive hepatology agenda for MASLD [4,7] by identifying combinations of food and microbial features that warrant prospective dietary or microbiome-targeted intervention testing in the older adult population. We emphasize that the analysis is intended to characterize joint diet-microbiome co-variation and its relation to hepatic steatosis at the population level; the results have no predictive utility at the individual level and are not intended as a clinical prediction tool.

## 2. Methods

### 2.1. Study Population

Previous studies in the area have demonstrated a distinctive rural-dwelling gut microbiota profile relative to the urban Kyoto residents of the same age and sex [20] and characterized the relationship between nutrient and food intake, dietary diversity, and gut microbiota in the older community residents with respect to frailty [21]. Between 20 July 2017 and 31 December 2021, 786 individuals provided fecal samples, completed dietary and lifestyle questionnaires, and underwent venous blood sampling. To restrict the analysis to non-alcoholic liver phenotypes, 85 participants exceeding the conventional heavy-drinking threshold (≥210 g pure ethanol per week for men, ≥140 g per week for women) were excluded, yielding the analytic cohort of *n* = 701 (244 men, 457 women). The threshold was adopted by the 2024 EASL–EASD–EASO clinical practice guidelines [2] and the 2026 Japanese evidence-based clinical practice guidelines for MASLD [6] to distinguish between MASLD and MetALD/ALD.

Comorbidities including type 2 diabetes mellitus (based on self-reported physician diagnosis, current use of glucose-lowering medications, or fasting plasma glucose ≥ 126 mg/dL and/or HbA1c ≥ 6.5%), hypertension (self-report, current antihypertensive medication, or blood pressure ≥ 140/90 mmHg), and dyslipidemia (self-report, current lipid-lowering medication, or triglyceride ≥ 150 mg/dL or HDL-cholesterol ≤ 40 mg/dL in male, ≤50 mg/dL in female) were ascertained by trained public health nurses using a structured questionnaire linked to concurrent laboratory measurements. Habitual leisure-time physical activity was assessed by a single-item questionnaire adapted from the Japanese National Health and Nutrition Survey, with ‘habitual exercise’ defined as engagement in any regular leisure-time activity for at least 30 min at a time, twice per week, sustained for at least one year.

The study conformed to the ethical principles of the Declaration of Helsinki, and the research protocol was approved by the Ethics Committee of Kyoto Prefectural University of Medicine (approval number: ERB-C-885-9). All participants provided written informed consent prior to enrollment. This study was registered with the UMIN Clinical Trials Registry (UMIN-CTR), UMIN000019486, on 1 December 2015.

### 2.2. Dietary Assessment

Habitual dietary intake during the month preceding the study was assessed using the brief-type self-administered diet history questionnaire (BDHQ), a validated 58-item food-frequency instrument widely used in Japanese epidemiological studies [23,24]. Although the BDHQ is designed for self-completion, trained public health nurses (hokenshi) at the survey sites supported each participant to maximize response accuracy and minimize missing data in this older adult population as they were all 65 years or older. Support included clarifying item wording, reviewing recall of portion sizes and frequencies, and verifying that all items had been answered. Estimated intake of 70 food items was adjusted for total energy intake using the density method (per 1000 kcal) and standardized to z-scores. To improve interpretability and stability of pattern extraction, all items were aggregated into 31 food groups by nutritional and culinary similarity (Supplementary Table S1). Three seasonal duplicate items (Citrus, Persimmons, Strawberries seasonal) were removed.

### 2.3. Analysis of the Fecal Microbiota

Fecal microbial DNA was extracted with a Maxwell^®^ RSC Fecal Microbiome DNA Kit (Promega, Tokyo, Japan) according to the manufacturer’s instructions. The V3–V4 region of the 16S rRNA gene was amplified using the primer set consisting of 341F (5′-CCTACGGGNGGCWGCAG-3′) and 805R (5′-GACTACHVGGGTATCTAATCC-3′) with Tks Gflex DNA Polymerase (TaKaRa Bio, Kusatsu, Japan). Polymerase chain reaction (PCR) amplification was performed using the following thermal cycling conditions: an initial denaturation at 95 °C for 3 min, followed by 25 cycles consisting of denaturation at 95 °C for 30 s, annealing at 55 °C for 30 s, and extension at 72 °C for 30 s, concluding with a final elongation step at 72 °C for 5 min. The resulting amplicons were purified using NucleoFast96 PCR plates (TaKaRa Bio), and a subsequent indexing PCR was carried out using unique dual-index primer sets compatible with MiSeq sequencing, following Illumina’s standard protocol (Illumina, San Diego, CA, USA). After indexing, PCR products were purified, normalized using the SequalPrep Normalization Plate Kit (Life Technologies, Tokyo, Japan), and pooled at equimolar concentrations. The pooled library was purified using AMPure XP magnetic beads (Beckman Coulter, Brea, CA, USA). The resulting purified library was subjected to 285-bp paired-end sequencing on the Illumina MiSeq platform using the MiSeq Reagent Kit v3 (Ilumina Inc., San Diego, CA, USA). Data obtained from MiSeq sequencing were analyzed as described by Miura et al. [25], with some exceptions. The QIIME2 [26] version 2024.5 was used, and the taxonomy of amplicon sequence variants was assigned against SILVA 138 [27]. The raw 16S rRNA gene sequencing data generated in this study have been deposited in the NCBI Sequence Read Archive under accession numbers PRJNA1470357 and PRJNA1440982; the corresponding subject-level identifier list (Microbiota-ID, age, sex, FLI, and FIB-4) is provided in Supplementary Table S2. All other relevant data supporting the findings of this study are included within this paper and its Supplementary Information Files.

The relative abundance data at the genus level were transformed using a centered log-ratio (CLR) transformation [28] to account for compositionality. All microbiota analyses were performed using CLR-transformed and z-standardized genus-level data.

### 2.4. Hepatic Phenotypes and the FIB-4 Age-Residualization

FLI was calculated as

FLI = (e^L^)/(1 + e^L^) × 100

where [8]

L = 0.953 × ln(TG) + 0.139 × BMI + 0.718 × ln(γGT) + 0.053 × waist circumference − 15.745.

The log-transformed FLI was z-scored as Log[FLI]_Z.

FIB-4 = (age × AST)/(platelet × √ALT)

the natural log was z-scored as Log[FIB-4]_Z [9]. The age term in the numerator of the formula of FIB-4 explains 21.3% of the variance in Log[FIB-4]_Z of the studied elderly cohort (β = 0.078 per year, *p* < 10^−37^). To prevent over-adjustment due to age as a covariate, we regressed Log[FIB-4]_Z on age and used the resulting residual, re-standardized, as Log[FIB-4]_age-resid_Z. This variable is by construction orthogonal to age (Pearson r = 0.000) and represents the ‘age-independent’ fibrosis signal (Supplementary Figure S1).

For clinical context, the FLI ranges from 0 to 100; established categorical thresholds are FLI < 30 (steatosis excluded), 30–59 (indeterminate), and 60 (steatosis likely) [8]. FIB-4 categorical thresholds in the general adult population are FIB-4 < 1.3 (advanced fibrosis excluded), 1.3–2.67 (indeterminate), and >2.67 (advanced fibrosis likely) [9]. Because FIB-4 rises linearly with age, an age-adjusted cutoff of 2.0 has been proposed for individuals aged ≥ 65 years. In this study, hepatic steatosis was defined by the surrogate FLI index rather than by imaging (ultrasound, MRI-PDFF) or histology, and this design choice is discussed as a limitation. FLI and FIB-4 were retained as continuous outcomes throughout our primary analyses to maximize statistical power.

### 2.5. Statistical Analysis

All analyses were performed in Python 3.10 (scikit-learn 1.4, statsmodels 0.14, factor_analyzer 0.5, scipy 1.13) and R 4.3. Two-sided α = 0.05 was used unless otherwise noted.

Dietary and microbiome pattern extraction. We applied the Varimax-rotated principal component analysis (PCA) separately to the 31 food groups and 47 genera, retaining four factors each based on scree-plot inflection, eigenvalue >1, cumulative variance, and biological interpretability. Subject-level factor scores were submitted to two-stage clustering (Ward hierarchical linkage to provide centroids for k-means) with k = 2–6 evaluated; k = 4 was chosen for both modalities based on a combined evaluation of the silhouette coefficient, Calinski–Harabasz index, biological interpretability of factor loadings, and bootstrap cluster stability assessed by the Jaccard similarity (B = 100; per-cluster Jaccard 0.57–0.78 for food clusters and 0.50–0.75 for microbiome enterotypes, with averages of 0.69 and 0.61, respectively). Diagnostic plots and the underlying values are provided in Supplementary Figure S3 and Supplementary Table S3.

Diet–microbiome synchronization. We performed canonical correlation analysis (CCA) on the 31 × 47 food–microbe matrix to identify the pairs of latent axes maximally correlated between modalities. Statistical significance of the canonical correlations was assessed by permutation (B = 100; null distribution generated by random row-shuffling of one matrix).

Joint diet–microbiome prediction of liver phenotypes. We applied multi-response partial least squares (PLS) regression—equivalent to RRR for a multivariate response—with the 78-dimensional concatenated predictor (31 food + 47 microbe) and two responses (Log[FLI]_Z and Log[FIB-4]_age-resid_Z), extracting six components. We compared in-sample R^2^ of the joint model with diet-only and microbiome-only PLS. Predictive performance was estimated by repeated stratified 10-fold cross-validation (5 repeats), and statistical significance of the in-sample fit was assessed by Y-permutation test (B = 500). Y-loadings (the contribution of each PLS component to each response) were used to attribute the components to either FLI or FIB-4 phenotype. The number of PLS components (six) was determined a priori as the maximum sufficient to capture both FLI-dominant and FIB-4-dominant signal (PLS1–PLS3) plus their orthogonal complements, subject to the parsimony constraint that no additional component provided a >1% gain in cross-validated Q^2^ (5-fold; predefined stopping rule). Cumulative variance explained by the six components in the 78-feature predictor block was 51.2% (individual components: 15.8%, 11.4%, 8.9%, 6.3%, 4.8%, 4.0%); cumulative variance explained in the two-outcome response block was 12.4% (dominated by PLS1 for FLI at 8.1% and PLS3 for FIB-4-residualized at 2.9%). Because the primary purpose of the model is a descriptive characterization of joint diet-microbiome co-variation rather than out-of-sample prediction, the interpretation of any given axis is based on loading structure and permutation-based significance rather than on cross-validated R^2^.

Mediation analysis. Bootstrap (B = 500) mediation was conducted with the diet PLS1 score as exposure, microbiome PLS1 score as mediator, and Log[FLI]_Z (or Log[FIB-4]_age-resid_Z) as outcome. The indirect effect (a × b) and mediated proportion were reported with bias-corrected 95% confidence intervals.

Pair-wise food–genus correlations. Partial Spearman correlations adjusted for age and sex were computed for all 31 × 47 = 1457 food–genus pairs, with Benjamini–Hochberg correction at false discovery rate (FDR) q < 0.05.

Covariate adjustment. To avoid over-adjustment of the variables embedded in FLI or FIB-4 calculations, we excluded BMI, waist circumference, triglycerides and γGT from FLI models, and AST, ALT and platelet count from FIB-4 models; age was excluded from FIB-4 models because residualization had already removed it. The retained adjustment variables included sex, HbA1c, diagnosed diabetes mellitus, hypertension, current smoking, habitual exercise, depression, frailty (frailty index ≥ 0.21 by Searle criteria [21]), sarcopenia (AWGS 2025 criteria [29]), multimorbidity (≥2 chronic diseases), and polypharmacy (≥5 medications). BMI was retained as a covariate for FIB-4 models, as it is not incorporated in the FIB-4 calculation.

## 3. Results

### 3.1. Participant Characteristics

The analytic cohort comprised 701 community-dwelling older adults (35% men, mean age 73.1 ± 5.9 years, range 65–101). Mean BMI was 22.95 ± 2.99 kg/m^2^ and waist circumference 84.0 ± 8.9 cm. The low percentages of diabetes (10.4%), hypertension (38.1%), dyslipidemia (30.5%), multimorbidity (40.4%), current smoking (25.7%), habitual exercise (42.4%), depression (22.5%), frailty (15.8%), sarcopenia (8.0%) and polypharmacy (6.3%) reflected a relatively healthy aging Japanese community (Table 1). FLI was available for 697 participants (Log[FLI]_Z: mean −0.06 ± 0.99) and FIB-4 for all 701 (Log[FIB-4]_Z: mean −0.005 ± 0.995). Among 73 participants (10.4%) with type 2 diabetes mellitus, 62 (84.9%) were receiving pharmacological glucose-lowering treatment (oral hypoglycemics and/or insulin). Median FLI in the analytic cohort was 12.8 (IQR: 5.9–27.4), and the categorical distribution was FLI < 30 in 448 (64.3%), 30–59 in 204 (29.3%), and ≥ 60 in 45 (6.4%) participants. Median FIB-4 was 1.71 (IQR: 1.35–2.20); using the age-adjusted ≥ 65-year cutoff of 2.0, 220 (31.4%) participants were classified as at increased risk of advanced fibrosis. Habitual leisure-time exercise (≥2 sessions/week for ≥30 min sustained for ≥ 1 year) was reported by 297 (42.4%) participants; the median self-reported total physical-activity time was 60 min/week.

### 3.2. Dietary Pattern and Microbiome Enterotype Identification

**Figure 1:**
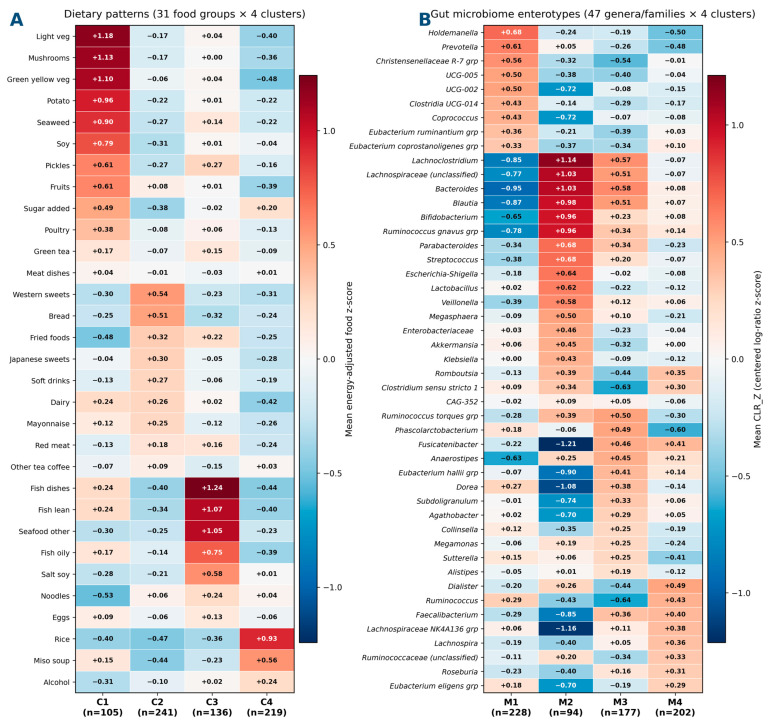
Identification of dietary patterns and gut microbiome enterotypes. (**A**) Heatmap of mean energy-adjusted z-scores of 31 food groups across the four dietary clusters (C1: Vegetable-Soy-Mushroom, *n* = 105; C2: Bread-Sweets-Fried foods, *n* = 241; C3: Seafood-Noodles-Salt, *n* = 136; C4: Rice-Miso soup-Alcohol, *n* = 219). Rows sorted by cluster of maximum loading. (**B**) Heatmap of mean centered log-ratio (CLR_Z) values of 47 genera across the four microbiome enterotypes (M1: *Prevotella*-*Christensenellaceae*, *n* = 228; M2: *Lachnoclostridium*-*R. gnavus*, *n* = 94; M3: *Bacteroides*-*Fusicatenibacter*, *n* = 177; M4: *Ruminococcus*-*Faecalibacterium*, *n* = 202). Cluster names follow a material-centric convention based on the most enriched components.

The dietary and gut microbial composition of the cohort was characterized by deriving discrete clusters using the Varimax-rotated PCA on the 31 food groups and 47 genera separately, followed by Ward + k-means clustering (k = 4), Calinski–Harabasz index, biological interpretability, and bootstrap Jaccard stability (B = 100; Supplementary Figure S3 and Supplementary Table S3). Cluster names follow a material-centric naming convention based on the food groups (mean energy-adjusted z-score) or the most enriched genera (mean CLR_Z) in each cluster relative to the overall mean (Figure 1). The k = 4 solution was selected primarily on biological interpretability grounds: although the Calinski-Harabasz statistic was maximal at k = 2, the k = 2 partition collapsed distinct dietary and microbial signals into overly broad clusters that obscured the identifiable washoku, vegetable/soy/dairy, seafood-rich, and noodle/processed-carbohydrate patterns of nutritional relevance in this elderly Japanese cohort. In sensitivity analyses (Supplementary Figure S3), we compared k = 2, 3, 4, and 5 solutions and confirmed that (i) the four-cluster solution maintained silhouette coefficients in the acceptable range (0.201 for diet, 0.203 for microbiome), (ii) the qualitative directions of cluster loadings on FLI were consistent at k = 3 and k = 4, and (iii) k = 5 produced an unstable smallest cluster (*n* < 40) with no additional biological information. We therefore prioritized biological interpretability and parsimony over the purely statistical optimum.

The four dietary clusters were: (C1) Vegetable-Soy-Mushroom (*n* = 105), which included light vegetables (mean z = +1.17), mushrooms (+1.13), green/yellow vegetables (+1.10), potato (+0.96), seaweed (+0.90), and soy (+0.79); (C2) Bread-Sweets-Fried foods (*n* = 241), which included western sweets (+0.54), bread (+0.51), fried foods (+0.32), Japanese sweets (+0.30), soft drinks (+0.27), and dairy (+0.26); (C3) Seafood-Noodles-Salt (*n* = 136), which included fish dishes (+1.24), lean fish (+1.07), other seafood (+1.05), oily fish (+0.75), and salt/soy condiments (+0.58); and (C4) Rice-Miso soup-Alcohol (*n* = 219), which included rice (+0.93), miso soup (+0.56), alcohol (+0.24), and sugar (+0.20).

The four microbiome enterotypes were: (M1) *Prevotella*-*Christensenellaceae* (*n* = 228): *Christensenellaceae* R-7 (mean CLR_Z = +0.60), *Prevotella* (+0.60), UCG-005 (+0.53), UCG-002 (+0.52), and *Clostridia* UCG-014 (+0.45) that are enriched for fiber-fermenting Firmicutes alongside *Prevotella*; (M2) *Lachnoclostridium*-*Ruminococcus gnavus* (*n* = 94): *Lachnoclostridium* (+1.14), *Bacteroides* (+1.00), *Bifidobacterium* (+0.99), *Lachnospiraceae* unclassified (+0.99), *Blautia* (+0.96), and *R. gnavus* group (+0.93) that are characterized by proteolytic and inflammation-associated taxa; (M3) *Bacteroides*-*Fusicatenibacter* (*n* = 177): *Bacteroides* (+0.56), *Lachnoclostridium* (+0.56), *Blautia* (+0.50), *Ruminococcus torques* group (+0.48), and *Lachnospiraceae* (+0.47), which is a mixed *Bacteroidetes*-*Lachnospiraceae* profile that discriminates from M2 by lower *R. gnavus* group enrichment; and (M4) *Ruminococcus*-*Faecalibacterium* (*n* = 202): *Dialister* (+0.52), *Ruminococcus* (+0.45), *Faecalibacterium* (+0.41), *Fusicatenibacter* (+0.40), *Lachnospiraceae* NK4A136 (+0.38), and *Ruminococcaceae* (+0.36) that are, dominated by short-chain fatty acid–producing commensals.

Importantly, assignments of the dietary cluster and microbiome enterotype were statistically independent at the category level (χ^2^ = 6.82, df = 9, *p* = 0.66; Cramér V = 0.05), and all 16 cells of the joint 4 × 4 mosaic contained subjects (smallest cell *N* = 9, largest *N* = 74), enabling stratified inspection.

### 3.3. Diet × Microbiome Cluster Mosaic and the Limits of Discrete Partitioning

**Figure 2:**
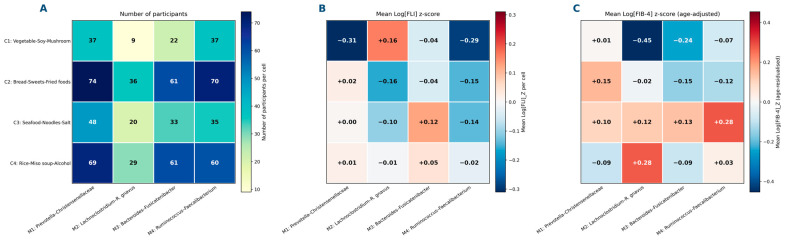
Diet × microbiome 16-cell mosaic. (**A**) Number of subjects per combination; (**B**) mean Log[FLI]_Z per cell; (**C**) mean Log[FIB-4] age-residualized Z per cell. The four diet (rows) and four microbiome (columns) clusters are statis-tically independent (χ^2^ = 6.82, *p* = 0.66), supporting that diet and gut microbiome capture non-redundant infor-mation. Two-way ANCOVA did not detect significant main effects or their interaction on either outcome (all *p* > 0.10).

We then examined the extent to which the discrete 4 × 4 cross-tabulation of dietary clusters and microbiome enterotypes explained the variation in the hepatic phenotypes. The mosaic visualization is shown in Figure 2. The lowest mean FLI was observed in Vegetable-Soy-Mushroom × *Prevotella*-Christensenellaceae (−0.31) and Vegetable-Soy-Mushroom × *Ruminococcus*-*Faecalibacterium* (−0.29), whereas the lowest mean FIB-4_resid was in Vegetable-Soy-Mushroom × *Lachnoclostridium*-*R. gnavus* (−0.51), suggesting an additive but not multiplicative co-occurrence of protective patterns (Figure 2). However, two-way anlysis of covariance revealed no significant main effects for either categorical assignment or their interaction on either outcome (all *p* > 0.10). Thus, hard partitioning of either modality—and even their joint cross-tabulation—did not yield statistically detectable group differences in FLI or FIB-4_resid, motivating the use of continuous multivariate methods described in the following sections.

### 3.4. Diet–Microbiome Synchronization (CCA)

**Figure 3:**
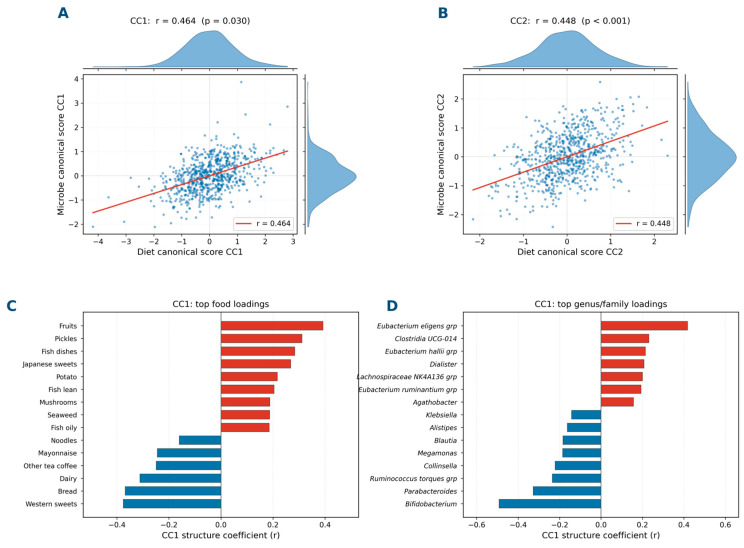
Diet–microbiome synchronization revealed by canonical correlation analysis (CCA). (**Top**) Scatter plots of the diet (*x*-axis) and microbe (*y*-axis) canonical scores for canonical pair 1 (CC1, r = 0.464, *p* = 0.030 by permutation) and canonical pair 2 (CC2, r = 0.448, *p* < 0.001), with the linear regression fit (red line) and marginal kernel density estimates for each score distribution (blue shaded curves) displayed alongside each panel. Each blue point represents one participant (*n* = 701). The positive slopes and significant canonical correlations demonstrate substantial multivariate co-variation between habitual dietary patterns and gut microbial composition in the Kyotango cohort. (**Bottom**): Structure coefficients for the top 12 foods (**left**) and genera (**right**) on canonical axis 1. Fruits and *Eubacterium eligens* group are highlighted (bold, dark-blue) as the top positive loadings on the food and genera sides, respectively, of CC2.

**Figure 4:**
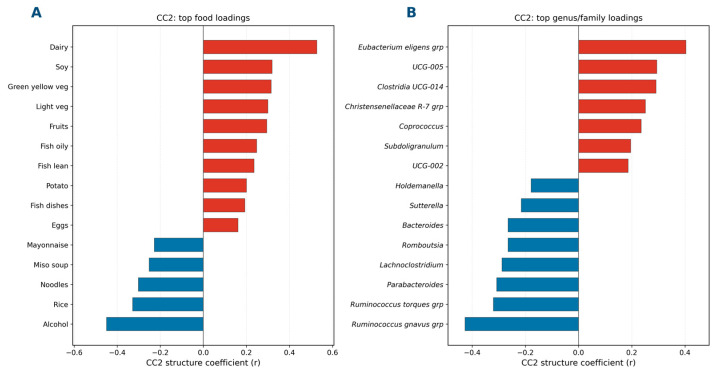
Structure coefficients for canonical axis 2 structure coefficients. (**A**): foods (top 15); (**B**): genera (top 15). CC2 separates a dairy/soy/vegetable/fish/fruits-rich axis (positive) paired with *E. eligens*/*Christensenellaceae*/UCG-005 (positive) from an alcohol/rice/noodles/miso-rich axis (negative) paired with *R. gnavus* group/*R. torques* group/*Bacteroides/Lachnoclostridium* (negative).

Despite the categorical independence of the discrete cluster assignments, we hypothesized that the underlying continuous variation in diet and microbiome composition may be synchronous at the multivariate level. We therefore applied CCA to the 31 food × 47 genus matrix. All four pairs reached statistical significance by permutation testing (Table 2); the first two canonical pairs are visualized in Figure 3: canonical r = 0.464, 0.448, 0.427, 0.396 (all *p* ≤ 0.030). The first canonical axis (CC1) loaded positively on the diet side with fruits, pickles, fish dishes, Japanese sweets, potato, lean fish, mushrooms, seaweed and fish (oily)—a ‘traditional Japanese plant + fish’ axis—and negatively on western sweets, bread, dairy, tea/coffee, mayonnaise, noodles, red meat and soft drinks. The corresponding microbiome side loaded positively on *Eubacterium eligens* group, *Clostridia* UCG-014, *Eubacterium hallii* group and *Dialister* (fiber-fermenting Firmicutes), and negatively on *Bifidobacterium*, *Parabacteroides*, *Collinsella* and *Ruminococcus torques* group. The second canonical axis (CC2) discriminated dairy + soy + vegetables + fish + fruits (positive) from alcohol + rice + noodles + miso soup (negative) on the diet side, paired with *E. eligens* + UCG-005 + *Christensenellaceae* R-7 + *Clostridia* UCG-014 (positive) vs. *R. gnavus* group + *R. torques* group + *Parabacteroides* + *Lachnoclostridium* + *Romboutsia* + *Bacteroides* (negative) on the microbiome side. The detailed structure coefficients for CC1 and CC2 are shown in Figure 3 and Figure 4.

### 3.5. Joint Diet–Microbiome Prediction of FLI and FIB-4

**Figure 5:**
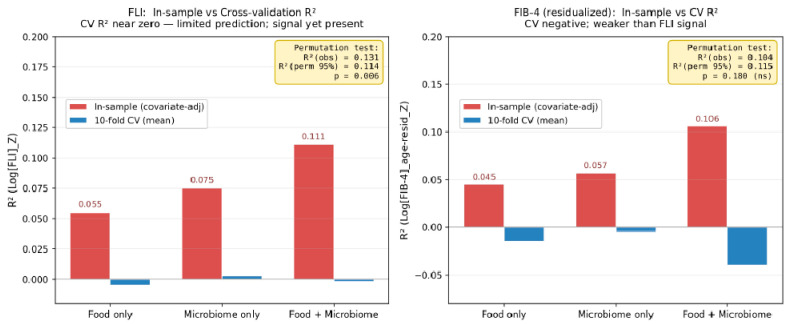
R^2^ for the diet, microbiome, and integrated models for FLI (**left**) and FIB-4 residualized (**right**). In-sample R^2^ (red) approximately doubles in the integrated model. Cross-validation R^2^ (blue) is near zero, indicating limited generalization. Permutation-test *p* values are shown in the panels.

We then evaluated whether an integrated modeling of diet and microbiome can provide a better prediction for liver phenotypes than either modality alone. After covariate adjustment, in-sample R^2^ for the diet-only PLS was 0.055 (FLI) and 0.045 (FIB-4_resid), while the microbiome-only PLS achieved 0.075 and 0.057, respectively. The combined diet + microbiome model approximately doubled the explanatory power: R^2^(FLI) = 0.111 and R^2^(FIB-4_resid) = 0.106 (Figure 5; Table 3); these covariate-adjusted values are the primary in-sample R^2^ estimates reported throughout this paper (age, sex, HbA1c, diabetes mellitus, hypertension, smoking, habitual exercise, depression, frailty, sarcopenia, multimorbidity and polypharmacy were partialled out from the outcomes before fitting the PLS model). Because Y-permutation testing operates by re-shuffling the outcome vector and therefore neutralises any pre-adjustment, the significance test itself was performed on the unadjusted (raw) in-sample R^2^ of the same combined model. This unadjusted R^2^ was slightly higher, as expected: unadjusted R^2^_observed(FLI) = 0.131 vs permutation 95th percentile = 0.114 (*p* = 0.006), whereas unadjusted R^2^_observed(FIB-4) = 0.104 vs 0.115 (*p* = 0.180). The covariate-adjusted R^2^ (0.111/0.106) and the unadjusted R^2^ (0.131/0.104) refer to the same fitted model—the ~0.02 gap for FLI reflects the small independent explanatory contribution of the sociodemographic and cardiometabolic covariates. Repeated 10-fold cross-validation indicated that the out-of-sample R^2^ was near zero or marginally negative for all models, reflecting that the multivariate signal, while statistically detectable in-sample, does not yet support quantitative prediction at the individual level. We therefore interpret the proposed integrated model as a tool for biological pattern interpretation, not personalized prediction.

### 3.6. Multi-Response RRR Identifies FLI- and FIB-4-Dominant Axes

**Figure 6:**
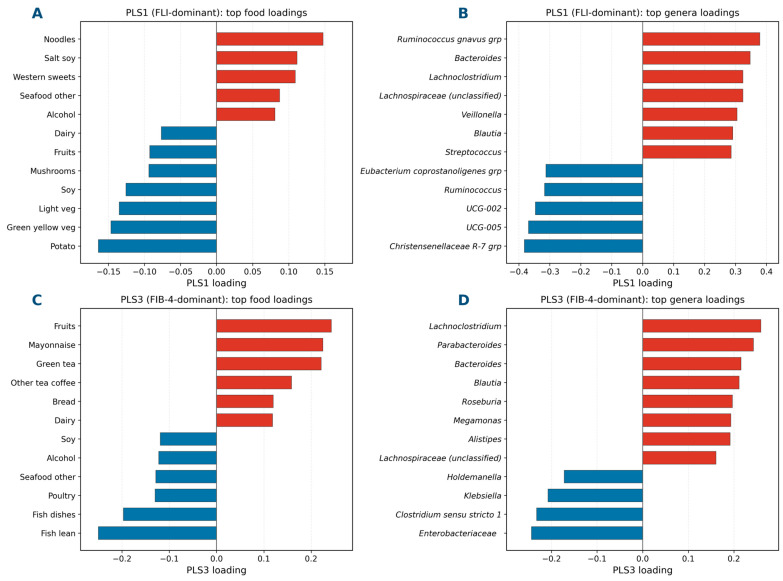
Top loadings on FLI-dominant PLS1 (**top row**) and FIB-4-dominant PLS3 (**bottom row**). Red bars: positive loading; blue bars: negative. Food groups on the (**left**), genera on the (**right**).

To disentangle the diet–microbiome patterns specific to steatosis and fibrosis, we examined the Y-loadings of the six PLS components extracted per the model specification in Section 2.5. Of these six components, PLS1 was FLI-dominant (Y_FLI = 0.109, Y_FIB-4 = 0.033), PLS3 was FIB-4-dominant (Y_FLI = 0.064, Y_FIB-4 = −0.224), and both FLI and FIB-4 contributed to PLS2/PLS4. The biological content of each axis (Figure 6) was as follows.

PLS1 (FLI-dominant): Higher PLS1 scores corresponded to higher Log[FLI]_Z. The diet side loaded positively on noodles, and negatively on green/yellow vegetables, light vegetables, potato, mushrooms, and soy. The microbiome side loaded positively on *Ruminococcus gnavus* group, *Lachnoclostridium*, *Bacteroides*, *Lachnospiraceae* (unclassified genus), *Streptococcus*, *Veillonella*, *Bifidobacterium*, *Escherichia*–*Shigella*, and *Megasphaera*—taxa consistent with a pro-inflammatory/proteolytic profile—and loaded negatively on all fiber-fermenting Firmicutes, such as *Christensenellaceae* R-7 group, UCG-005, UCG-002, *Ruminococcus*, *Eubacterium coprostanoligenes* group, *Coprococcus*, and *Clostridia* UCG-014.

PLS3 (FIB-4-dominant): Higher PLS3 scores corresponded to lower Log[FIB-4]_age-resid_Z. The diet side loaded positively (fibrosis-protective) on fruits, light vegetables, mayonnaise, green tea, green-yellow vegetables, and dairy, and negatively on alcohol and noodles. The microbiome side loaded fibrosis-protected on *Lachnoclostridium*, *Bacteroides*, *Blautia*, *Parabacteroides*, *Roseburia*, and unclassified *Lachnospiraceae*, and fibrosis-risk on *Holdemanella*, *Clostridium sensu stricto* 1, and *Enterobacteriaceae* (unclassified genus).

### 3.7. Gut Microbiome Statistically Accounts for Part of the Diet-FLI Association

**Figure 7:**
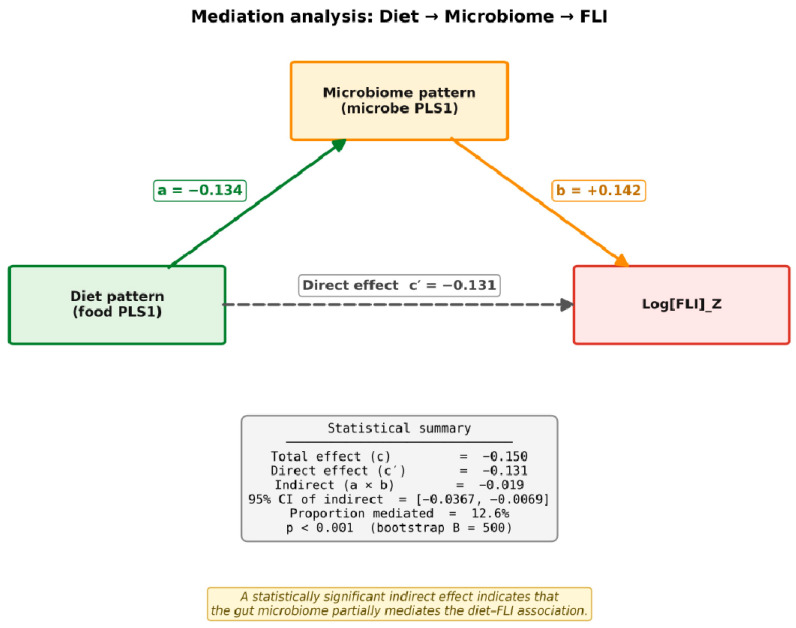
Mediation diagram for diet → microbiome → FLI. The indirect effect (a × b) is statistically significant, accounting for 12.6% of the total diet–FLI association.

Bootstrap mediation analysis (B = 500) using the diet PLS1 score as exposure, microbiome PLS1 score as mediator, and Log[FLI]_Z as outcome (covariate-adjusted) revealed that 12.6% of the total dietary effect on FLI was indirectly transmitted through the gut microbiome (Figure 7). The path-a coefficient (diet → microbiome) was −0.134, path-b (microbiome → FLI | diet) was +0.142, total effect was −0.150, direct effect was −0.131, and indirect effect (a × b) was −0.019 with bootstrap 95% confidence interval excluding zero (−0.037 to −0.007; *p* < 0.001). In contrast, for Log[FIB-4]_age-resid_Z, only 1.2% of the total dietary effect was mediated by the microbiome (indirect effect = −0.002, 95% CI: −0.015 to +0.010, *p* = 0.69), indicating that the association between diet and age-independent fibrosis is mediated primarily through mechanisms other than large-scale shifts in the gut microbiome.

### 3.8. Pair-Wise Food–Genus Correlations (FDR-Corrected)

**Figure 8:**
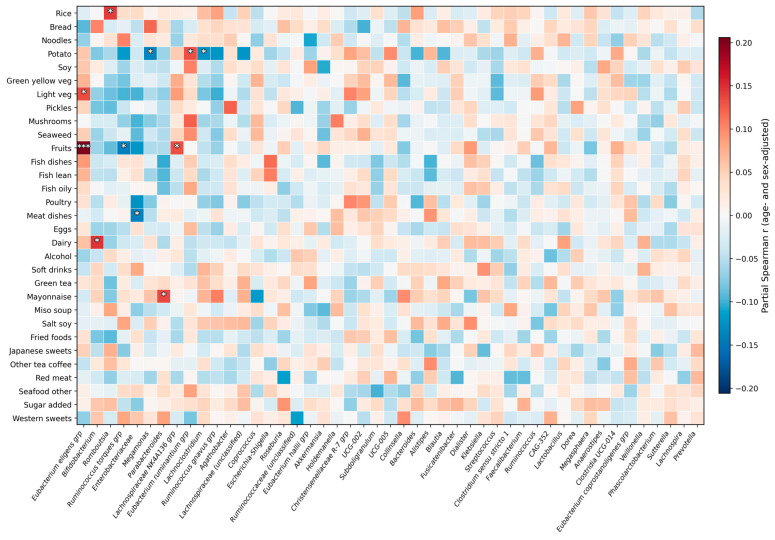
Heatmap of the age- and sex-adjusted partial Spearman correlation coefficient (r) between every food group (rows; *n* = 31) and every gut microbial genus/family (columns; *n* = 47)—i.e., all 1457 food × genus pairs. Cell colour encodes r (red = positive, blue = negative). Symbols in cells: *** marks the single food–genus pair that survived Benjamini–Hochberg (BH) multiple-testing correction at FDR q < 0.05 (Fruits × *Eubacterium eligens* group; r = +0.202, *p* = 7.2 × 10^−8^, q = 1.0 × 10^−4^, top-left cell), consistent with the well-established association between plant-fibre intake and this fibre-fermenting commensal. * marks the ten additional biologically interpretable pairs that reached an unadjusted *p* < 10^−3^ but did not survive BH-FDR correction (Dairy × *Bifidobacterium*; Rice × *Romboutsia*; Fruits × *Ruminococcus torques* group; Meat dishes × *Enterobacteriaceae*; Potato × *Megamonas*; Mayonnaise × *Parabacteroides*; Light vegetables × *Eubacterium eligens* group; Fruits × *Lachnospiraceae* NK4A136 group; Potato × *Eubacterium ruminantium* group; Potato × *Lachnoclostridium*). The sparse pattern (1 of 1457 pairs surviving FDR) is consistent with the broader microbiome literature at the population scale and reinforces our reliance on multivariate axes (CCA, PLS) rather than on isolated pair-wise associations.

Only one of the 1457 food–genus partial Spearman correlations (age- and sex-adjusted) survived Benjamini–Hochberg correction at FDR q < 0.05: the pair fruits × *Eubacterium eligens* group (r = +0.202, *p* = 7.2 × 10^−8^, q = 1.0 × 10^−4^; see Figure 8), consistent with the well-established association between plant-fiber intake and this fiber-fermenting commensal [30]. Several biologically interpretable pairs reached unadjusted *p* < 10^−3^ (dairy × *Bifidobacterium*; rice × *Romboutsia*; fruits × *R. torques* group; meat dishes × *Enterobacteriaceae*; potato × *Megamonas*; mayonnaise × *Parabacteroides*; Figure 8), but did not survive the FDR. Pair-wise FDR-significant associations with FLI were limited to *Escherichia–Shigella* (r = +0.135, q = 0.016), a lipopolysaccharide-producing taxon consistent with the endotoxin-driven steatosis hypothesis. No individual food–FLI or food/genus–FIB-4_resid associations survived the FDR correction, again pointing to the necessity of multivariate pattern analysis in this domain.

## 4. Discussion

In a Japanese community-dwelling cohort of 701 older adults free of heavy alcohol consumption, habitual dietary patterns and gut microbial composition co-varied measurably and jointly related to the fatty liver index (FLI). Importantly, our analysis is descriptive and hypothesis-generating rather than predictive: although in-sample permutation testing supported the multivariate diet-microbiome-FLI link (R^2^ = 0.111; *p* = 0.006), the corresponding cross-validated R^2^ was negative, indicating that the absolute effect sizes are modest and the model does not generalize as a clinical prediction tool at this sample size. We therefore frame our results as covariation patterns warranting prospective replication and intervention testing rather than as a predictive ecosystem model. Within this scope, three observations are noteworthy: (i) four dietary patterns and four microbial clusters were broadly independent at the discrete cluster level, yet continuous CCA axes captured robust co-variation; (ii) the diet-microbiome combination approximately doubled in-sample explained variance for FLI relative to either domain alone; and (iii) bootstrap mediation provided modest but non-zero evidence for a microbiota-mediated path from diet to hepatic steatosis.

### 4.1. Scope of Analysis and the Descriptive Framework

The classical ‘diet to liver’ epidemiological framework treats diet as a single exposure, while the ‘microbiome to liver’ framework focuses on microbial metabolites and bacterial translocation. We did not attempt to supplant either framework or to argue for a deterministic ‘ecosystem’ construct; rather, we asked whether the two domains, when modeled jointly, contribute non-redundantly to a single clinical surrogate (FLI) in an older community population. The doubling of in-sample explanatory power upon combining the two domains, the multivariate CCA structure, and the modest but bootstrap-supported mediated path are each compatible with a joint contribution, and they motivate prospective testing of integrated diet-and-microbiome nutritional strategies in preventive hepatology. We explicitly note that at the level of individual food-genus pairs only one of 1457 partial-Spearman correlations survived FDR correction; this is consistent with the broader microbiome literature at population scale and supports our reliance on multivariate axes rather than on isolated pair-wise signals.

### 4.2. Biological Interpretation of the FLI- and FIB-4-Dominant Axes

PLS1 (FLI-dominant) implicates a refined-carbohydrate-rich, vegetable-poor diet, in conjunction with a *Bacteroides*/*Lachnoclostridium*/*R. gnavus* group-dominated, *Christensenellaceae*-poor microbiome, as the steatosis-risk axis. *R. gnavus* group has been repeatedly linked to chronic inflammation, obesity and NAFLD [31,32,33], producing a glucorhamnan polysaccharide with TLR4-stimulatory activity [34]. *Christensenellaceae* and *Clostridia* UCG-005, members of the *Christensenellales*, are robustly associated with lean phenotype and metabolic health across populations [35,36] and respond to dietary fiber substrate. Conversely, traditional Japanese plant-based components—soy, vegetables, and mushrooms—appeared on the protective side of PLS1; soy is a well-documented isoflavone source with hepatoprotective activity [37]. The pattern is consistent with the gradual westernization of the Japanese diet, in which refined carbohydrates have progressively displaced vegetable and soy intake. It is important to note that *R. gnavus* is also a common commensal in healthy adults, and its putative pathogenicity in MASLD depends on the specific strain and its glucorhamnan expression phenotype. Genus-level 16S rRNA gene amplicon sequencing (V3–V4) cannot resolve these strain-level distinctions, and confirmation of an *R. gnavus* pathogenic contribution in this cohort would require shotgun metagenomics or strain-specific PCR.

Beyond the *R. gnavus* example, the genus-level 16S V3–V4 profile used here cannot distinguish pathogenic from commensal strains within the same genus more broadly (for example, different *Bacteroides* species have opposing metabolic effects on host lipid and bile-acid handling), and 16S data carry no direct functional information on short-chain fatty acid (SCFA) synthesis, bile acid modification, or lipopolysaccharide (LPS) production. Because our biological interpretation of the CCA and PLS axes explicitly invokes fiber fermentation, LPS-driven inflammation and TLR4-mediated hepatic signaling, the absence of shotgun metagenomic function calls and paired SCFA/bile-acid/LPS metabolomics is a real limitation of the present study.

The parallel FIB-4-dominant axis (PLS3) showed weaker statistical support (permutation *p* = 0.18) and is presented as exploratory. Nonetheless, the directional pattern was biologically coherent: moderate alcohol intake clustered with *Enterobacteriaceae* enrichment on the fibrosis-risk side, while polyphenol- and antioxidant-rich foods (fruits, green tea) clustered with *Lachnospiraceae* SCFA producers (e.g., *Roseburia*) on the fibrosis-protective side. These patterns are consistent with the gut-LPS-Kupffer cell-stellate cell activation hypothesis [38], the FXR/TGR5-mediated anti-fibrotic action of microbial SCFAs [39,40], and the catechin-mediated suppression of hepatic stellate cell activation [41,42]. Because clinically significant fibrosis is uncommon in this community-dwelling cohort without overt MASLD (see Section 4.3 and Section 4.5), these PLS3 findings should be interpreted as hypothesis-generating rather than conclusive.

Taken together, the top loadings on our latent axes are biologically coherent with three well-established gut microbial mechanisms of hepatic phenotype modulation. First, fiber fermentation and SCFA production: on the FLI-protective side of PLS1, high loadings for vegetables, soy and fruit co-occur with *Faecalibacterium*, *Christensenellaceae*, *Roseburia* and *Fusicatenibacter*, all of which are established butyrate or propionate-producing Firmicutes whose fermentation of dietary fiber supplies SCFAs implicated in improved insulin sensitivity, reduced hepatic de novo lipogenesis and preserved intestinal barrier function. Second, bile acid biotransformation: the noodle-and-refined-carbohydrate loadings on the FLI-risk side of PLS1 co-occur with *Bacteroides*, *Lachnoclostridium* and the *R. gnavus* group, taxa carrying broad bile-salt-hydrolase and 7-alpha-dehydroxylase capacity that reshape the circulating bile acid pool and antagonize FXR/TGR5 signaling in the gut-liver axis. Third, LPS-driven low-grade inflammation: the *R. gnavus* glucorhamnan and the PLS3 *Enterobacteriaceae* signal converge on TLR4-mediated hepatic inflammation, a mechanism repeatedly implicated in steatosis-to-steatohepatitis progression in both animal and human studies. These interpretations remain hypothesis-level: direct confirmation would require shotgun metagenomic function calls (KEGG pathway abundances for SCFA synthesis, bile acid biotransformation, LPS biosynthesis) and paired stool/serum metabolomics (SCFAs, primary and secondary bile acids, endotoxin/LBP), which were not available in this cohort. Interim functional prediction from 16S data using PICRUSt2 or Tax4Fun2 is a feasible next step to test the mapping of latent axes onto these pathways (Section 4.7).

### 4.3. The FIB-4 Age-Confounding Problem in Elderly Cohorts

FIB-4 includes age in its formula numerator (age is a known fibrosis risk factor), which is a limitation for studies in elderly cohorts. In our 65–101 year-old sample set, age alone explained 21% of FIB-4 variance (β = 0.078 per year), implying that any food–FIB-4 or microbe–FIB-4 association will be contaminated by age unless explicitly controlled. However, direct adjustment for age as a covariate double-corrects for the age component already inside FIB-4 (over-adjustment). Our residualization approach, in which the Log[FIB-4]_Z was first regressed against age, and the resulting residuals were used as the outcome variable without further age adjustment, represents the age-related confounding and follows the approach adopted in recent epidemiologic studies of NAFLD progression markers in geriatric populations. The age-independent FIB-4 signal showed only a non-significant trend (permutation *p* = 0.18), which we interpret cautiously. Critically, this is a community-dwelling cohort from a Kyotango longevity area rather than a population of patients with diagnosed MASLD or chronic liver disease; the prevalence of clinically significant hepatic fibrosis is therefore expected to be low, and the dynamic range of FIB-4 in our sample is correspondingly narrow. The weaker FIB-4 signal likely reflects this floor effect—the cohort simply does not contain enough fibrotic phenotype variance for diet/microbiome predictors to capture—rather than a true absence of diet–microbiome–fibrosis associations. Therefore, validation in MASLD-enriched cohorts with FibroScan or magnetic resonance elastography will be essential before drawing fibrosis-specific conclusions.

### 4.4. Predictive Validity and Out-of-Sample Performance

We emphasize that the multivariate diet-microbiome-FLI model achieved in-sample but not out-of-sample explanatory power: 5-fold cross-validated R^2^ was negative, while in-sample R^2^ was 0.111 (permutation *p* = 0.006). This gap reflects the high feature-to-sample ratio (78 predictors for 697 participants) and the inherently modest absolute effect sizes typical of population-level diet-microbiome associations. The findings should therefore be interpreted as hypothesis-generating signals of joint diet-microbiome co-variation associated with FLI, not as a clinically deployable predictor. For FIB-4 the analogous combined model was non-significant on permutation testing (*p* = 0.18) and is reported as a secondary exploratory analysis only; we draw no biological inference from the FIB-4 axes beyond noting their internal-cohort patterns. Three specific consequences follow: (i) the identified latent structures should not be interpreted as robust predictive signatures; (ii) the reported associations primarily describe covariance patterns between diet, microbiota and FLI rather than causal or predictive relationships; and (iii) external validation of any candidate diet-microbiome axis in an independent, ideally non-Japanese cohort is essential before any translational claim can be made.

### 4.5. Novelty of Joint Modeling and Translational Implications

Individually, the ingredients of our findings are not novel: adherence to a vegetable- and soy-rich pattern has long been associated with reduced hepatic steatosis, and higher relative abundance of the *R. gnavus* group has been linked to obesity and NAFLD in previous literature [31,32,33]. What the joint model contributes is (i) the quantitative demonstration that dietary and microbial variance components are non-redundant—combining the two domains approximately doubles the in-sample explained variance for FLI relative to either domain alone (11.1% versus 5.5% for diet-only and 7.5% for microbiome-only), and (ii) an integrated map of which combinations of dietary and microbial features co-occur at each end of the FLI spectrum, information that is not available from univariate or single-modality analyses. These observations translate into concrete hypotheses for intervention design that would not be identifiable from diet-only or microbiome-only studies. For example, the finding that *R. gnavus* enrichment co-occurs with a noodle- and refined-carbohydrate-rich, vegetable-poor pattern motivates evaluation of pattern-targeted dietary counseling (increased vegetable and soy intake) combined with prebiotic fiber supplementation aimed at expanding *Christensenellaceae* and *Roseburia* in individuals with high baseline *R. gnavus* abundance. Conversely, the fruits × *Eubacterium eligens* co-signature suggests that polyphenol-rich fruit intake and *Lachnospiraceae*-supportive prebiotic strategies (e.g., resistant starch, inulin-type fructans) might be co-deployed for maintenance of the protective co-signature in individuals with borderline FLI. All such applications are hypothesis-generating and require prospective controlled testing.

### 4.6. Strengths and Limitations

Strengths of the proposed model include the well-characterized Japanese longevity cohort with abundant covariates (lifestyle, social, mental, frailty/sarcopenia, comorbidities, polypharmacy); a validated dietary instrument (BDHQ) administered with support from trained public health nurses (hokenshi) at the survey sites, which is expected to maximize response accuracy and minimize missing data in this older adult population; 16S rRNA profiling at the genus level; and a sophisticated multi-method analytic framework (PCA + clustering, CCA, multi-response RRR, bootstrap mediation, FDR correction, cross-validation) that triangulates the diet–microbiome–liver triad. Limitations include the cross-sectional design, which precludes causal inference; FLI and FIB-4 being surrogate indices rather than gold-standard hepatic phenotyping; the modest cross-validated predictive performance; the lack of FSCA and bile acid quantification; and the restriction to non-heavy drinkers, which may underestimate the alcohol contribution. Importantly, because this is a mostly healthy community-dwelling cohort from a longevity area rather than a population with diagnosed MASLD, the prevalence of clinically significant hepatic fibrosis is low and the dynamic range of FIB-4 is correspondingly limited; the fibrosis-related findings are therefore exploratory and require validation in MASLD-enriched populations with imaging or histology. Additionally, FLI inherently contains BMI, waist, triglycerides and γGT, so the most interpretable portion of the FLI signal is the variance unexplained by these constituent components—i.e., the additional information contributed by the diet and gut microbiome. Beyond these general limitations, six specific points warrant explicit acknowledgement in the context of the present analysis. First, hepatic steatosis was defined by the surrogate FLI index; direct imaging (ultrasound, MRI-PDFF) or histological confirmation was not available, so the study evaluates associations with a biomarker of steatosis rather than with directly measured liver fat. Second, dietary intake was estimated by BDHQ self-report and, while BDHQ has been validated against 16-day weighed diet records in Japanese adults [23,24], no biomarker-level validation of dietary intake (for example, plasma phospholipid fatty acids for fat quality, urinary nitrogen for protein intake, or urinary potassium for vegetable and fruit intake) was performed in this cohort. Third, the study population represents a rural Japanese longevity region with distinctive dietary habits (rice- and vegetable-rich washoku), gut microbiota composition (elevated *Faecalibacterium*, distinct *Christensenellaceae* profile), lean body composition (mean BMI 22.9 kg/m^2) and metabolic risk profile relative to Western populations; the findings should therefore not be generalized beyond similar community-dwelling older East Asian populations without external validation. Fourth, mediation analysis was conducted on cross-sectional data and cannot establish temporal mediation; the reported indirect effect quantifies a statistical decomposition of association under strong assumptions (no unmeasured diet-microbiome-FLI confounding; correct model specification; no reverse causation from FLI to microbiome), all of which are unlikely to hold strictly in observational data. Fifth, the 16S V3–V4 amplicon design provides genus-level resolution only, without species-level or strain-level discrimination and without direct functional information on SCFA synthesis, bile acid modification, or LPS production. Sixth, because in-sample multivariate explanatory power did not generalize to out-of-sample prediction, the study has no individual-level predictive utility, and its conclusions apply at the population-descriptive level only.

### 4.7. Future Directions

Three promising directions for future investigation emerge from this study. First, prospective follow-up in this cohort will allow testing whether baseline joint diet-microbiome co-variation patterns associate with incident liver enzyme elevation, MASLD progression, or all-cause mortality. Second, shotgun metagenomics combined with fecal and serum metabolomics (especially SCFAs, bile acid profiles and LPS) will move from association to mechanism, enabling pathway-level RRR. Third, intervention trials targeting identified candidate genera (*Christensenellaceae* R-7, *Roseburia*, *Lachnoclostridium*, *Parabacteroides*) via dietary or probiotic strategies—and assessing FLI/FIB-4/elastography responses—will translate these observational findings into preventive hepatology practice. As an interim step before full shotgun metagenomics becomes available, functional prediction from 16S data using PICRUSt2 or Tax4Fun2 could be pursued as a supplementary analysis to derive putative KEGG pathway abundances (SCFA synthesis, bile acid biotransformation, LPS biosynthesis) and to test whether the diet-associated microbial axes identified here map onto these functional pathways. Third, external validation in independent elderly non-Japanese cohorts is essential to determine whether the co-variation patterns identified here reflect a general diet-microbiome-FLI structure or a Kyotango-specific one.

## 5. Conclusions

In a community-dwelling Japanese cohort of older adults, habitual dietary patterns and gut microbial composition co-vary in a measurable, multivariate manner and jointly relate to the fatty liver index. Combining the two domains approximately doubled in-sample explanatory power for FLI relative to either domain alone. Bootstrap mediation analysis on cross-sectional data suggested that the gut microbial composition statistically accounts for a small portion (~12.6%) of the observed diet-on-FLI association under the standard mediation assumptions. Because 5-fold cross-validated R^2^ was negative, the model has no individual-level predictive utility and should be interpreted as a descriptive characterization of population-level covariance patterns and as a source of testable hypotheses for future prospective work. External validation in independent, ideally non-Japanese cohorts is essential before any translational claim, and combined diet-and-microbiome nutritional strategies motivated by these covariance patterns should be evaluated in controlled intervention trials.

## Figures and Tables

**Table 1:** Characteristics of the analytic cohort (*n* = 701).

Characteristic	Value	Missing (*n*)
Number (men/women)	244/457	0
Age, years (mean ± SD)	73.1 ± 5.9 (range 65–101)	0
BMI, kg/m^2^	22.95 ± 2.99	0
Waist circumference, cm	84.0 ± 8.9	2
Triglycerides, mg/dL	121.0 ± 60.6	0
HDL-C, mg/dL	67.5 ± 17.2	0
HbA1c, %	5.77 ± 0.54	0
AST, U/L	23.8 ± 8.8	0
ALT, U/L	20.7 ± 11.0	0
γ-GTP, U/L	27.3 ± 23.6	0
Platelet, ×10^4^/μL	22.1 ± 5.0	0
Log[FLI]_Z	−0.06 ± 0.99	4
Log[FIB-4]_Z	−0.005 ± 0.995	0
FLI (raw), median (IQR)	12.8 (5.9–27.4)	4
FLI < 30 (steatosis excluded), *n* (%)	448 (64.3)	–
FLI 30–59 (indeterminate), *n* (%)	204 (29.3)	–
FLI ≥ 60 (steatosis likely), *n* (%)	45 (6.4)	–
FIB-4 (raw), median (IQR)	1.71 (1.35–2.20)	0
FIB-4 ≥ 2.0 (age-adjusted ≥65 y cutoff), *n* (%)	220 (31.4)	–
Diabetes mellitus, *n* (%)	73 (10.4)	0
On glucose-lowering medication, *n* (% of diabetics)	62 (84.9)	–
Hypertension, *n* (%)	267 (38.1)	0
Dyslipidemia, *n* (%)	214 (30.5)	0
Multimorbidity (≥2), *n* (%)	283 (40.4)	0
Current smoking, *n* (%)	180 (25.7)	1
Habitual exercise, *n* (%)	297 (42.4)	0
Depression, *n* (%)	158 (22.5)	0
Polypharmacy (≥5 drugs), *n* (%)	44 (6.3)	0
Frailty, *n* (%)	111 (15.8)	0
Sarcopenia, *n* (%)	56 (8.0)	0

Values are mean ± SD or *n* (%). BMI = body mass index; HDL-C = high-density lipoprotein cholesterol; AST = aspartate aminotransferase; ALT = alanine aminotransferase; γ-GTP = γ-glutamyl transpeptidase; FLI = fatty liver index; FIB-4 = Fibrosis-4 index. Dyslipidemia was defined according to the 2024 EASL–EASD–EASO clinical practice guidelines on the management of MASLD.

**Table 2:** Canonical correlation analysis of diet and microbiome.

Canonical Pair	Diet–Microbiome r	Perm 95%ile	*p*-Value	Interpretation
CC1	0.464	0.46	0.03	Traditional Japanese plant + fish vs. Western refined (sweets/bread/dairy)
CC2	0.448	0.433	<0.001	Dairy/Soy/Vegetables/Fruits vs. Alcohol/Rice/Noodles/Miso
CC3	0.427	0.411	0.01	Western drinks/sweets + Enterobacteriaceae vs. Japanese staples + fiber-fermenting Firmicutes
CC4	0.396	0.392	0.01	Traditional washoku (seafood/miso/noodle) vs. fresh-produce

Permutation test with B = 100 row-shuffles of the microbiome matrix. All canonical pairs reached statistical significance.

**Table 3:** Multi-response RRR/PLS performance (covariate-adjusted).

Model	R^2^ FLI (in-Sample)	R^2^ FIB-4_resid (in-Sample)	R^2^ 10-fold CV (FLI/FIB-4)
Diet only (31 food groups)	0.055	0.045	negative/negative
Microbiome only (47 genera)	0.075	0.057	negative/negative
Diet + Microbiome (combined)	0.111	0.106	negative/negative
Permutation *p* (in-sample)	0.006	0.18	—

PLS with 4 components. Permutation test: B = 500. CV: repeated 10-fold (5 repeats). Negative CV R^2^ indicates lack of out-of-sample generalization despite statistically significant in-sample fit for FLI.

## Data Availability

The raw 16S rRNA gene sequencing data generated in this study have been deposited in the NCBI Sequence Read Archive (SRA) under accession numbers PRJNA1470357 and PRJNA1440982. All other relevant data supporting the findings of this study are included within the article and its Supplementary Information Files.

## Supplementary Materials

The following supporting information can be downloaded at: https://www.mdpi.com/article/10.3390/nu18142300/s1, Figure S1: Age-residualization of Log[FIB-4]_Z (*n* = 701); Figure S2: Study flow diagram. From 786 community-dwelling residents of the Kyotango longevity cohort, 85 heavy drinkers were excluded (men > 210 g/wk, women > 140 g/wk ethanol), yielding the analytic cohort of *n* = 701. All participants had complete BDHQ dietary, 16S rRNA microbiome, FLI and FIB-4 data; Figure S3: Cluster derivation diagnostics for food groups (*n* = 31) and microbiome genera (*n* = 47). (A,B) Scree plots showing PCA eigenvalues with cumulative variance explained; the Kaiser criterion (eigenvalue > 1) supports retention of four factors for each modality. (C) Silhouette coefficient and Calinski–Harabasz index across k = 2–6 cluster solutions; k = 4 was chosen as the best balance of partition quality and biological interpretability. (D) Bootstrap cluster stability (B = 100) at k = 4, plotted as Jaccard similarity per cluster (mean ± SD). Reference lines correspond to the Hennig [43] interpretation thresholds (≥0.75 highly stable, 0.60–0.75 moderately stable, 0.50 borderline). Numerical values are provided in Supplementary Table S3. Table S1: Aggregation of BDHQ items into 31 food groups; Table S2: NCBI sample registration list (SRA accession: PRJNA1470357, PRJNA1440982. Table S3: Cluster derivation diagnostics.
