# Supplementary material for: Joint Dietary and Gut Microbial Profiling and the Fatty Liver Index in Community-Dwelling Older Japanese: A Cross-Sectional, Hypothesis-Generating Analysis from the Kyotango Longevity Study
Source: Nutrients. 2026 Jul 14;18(14):2300. doi: 10.3390/nu18142300 (PMC13415844; doi:10.3390/nu18142300)
Supplement: Supplementary file 1 [file nutrients-18-02300-s001.zip › Figure S2_cohort_flow.pdf]

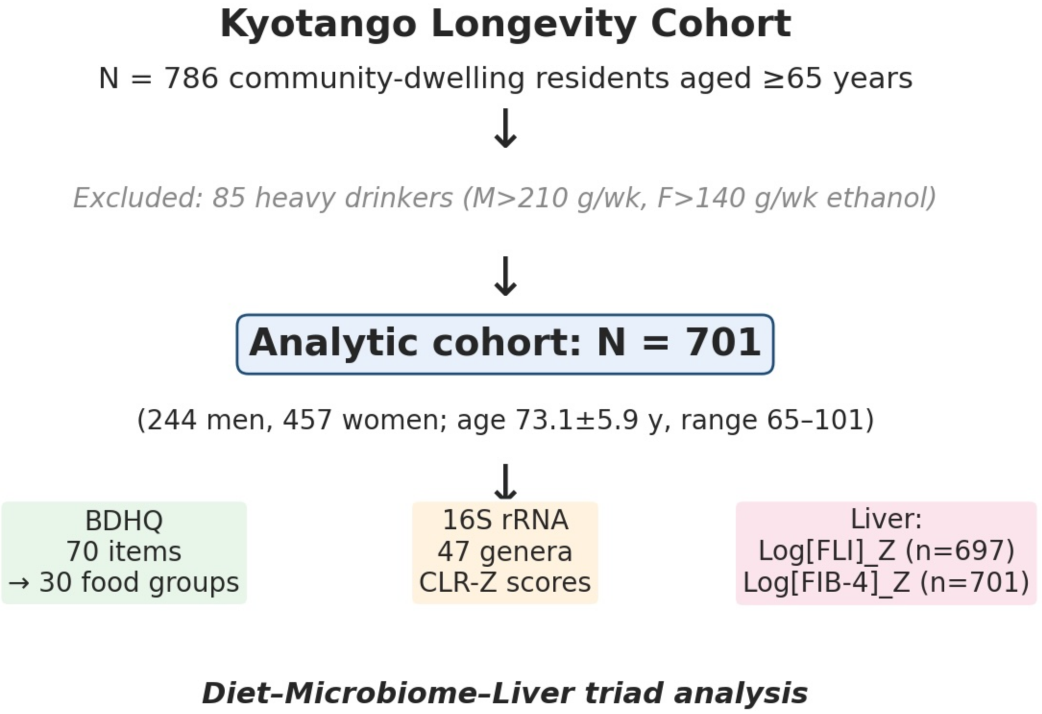
Kyotango Longevity Cohort
N = 786 community-dwelling residents aged ≥65 years
Excluded: 85 heavy drinkers (M>210 g/wk, F>140 g/wk ethanol)
Analytic cohort: N = 701
(244 men, 457 women; age 73.1±5.9 y, range 65–101)
BDHQ
70 items
→ 30 food groups
16S rRNA
47 genera
CLR-Z scores
Liver:
Log[FLI]_Z (n=697)
Log[FIB-4]_Z (n=701)
Diet–Microbiome–Liver triad analysis
