# Supplementary material for: Joint Dietary and Gut Microbial Profiling and the Fatty Liver Index in Community-Dwelling Older Japanese: A Cross-Sectional, Hypothesis-Generating Analysis from the Kyotango Longevity Study
Source: Nutrients. 2026 Jul 14;18(14):2300. doi: 10.3390/nu18142300 (PMC13415844; doi:10.3390/nu18142300)

Supplementary Figure S3. Cluster derivation diagnostics for food groups and microbiome enterotypes

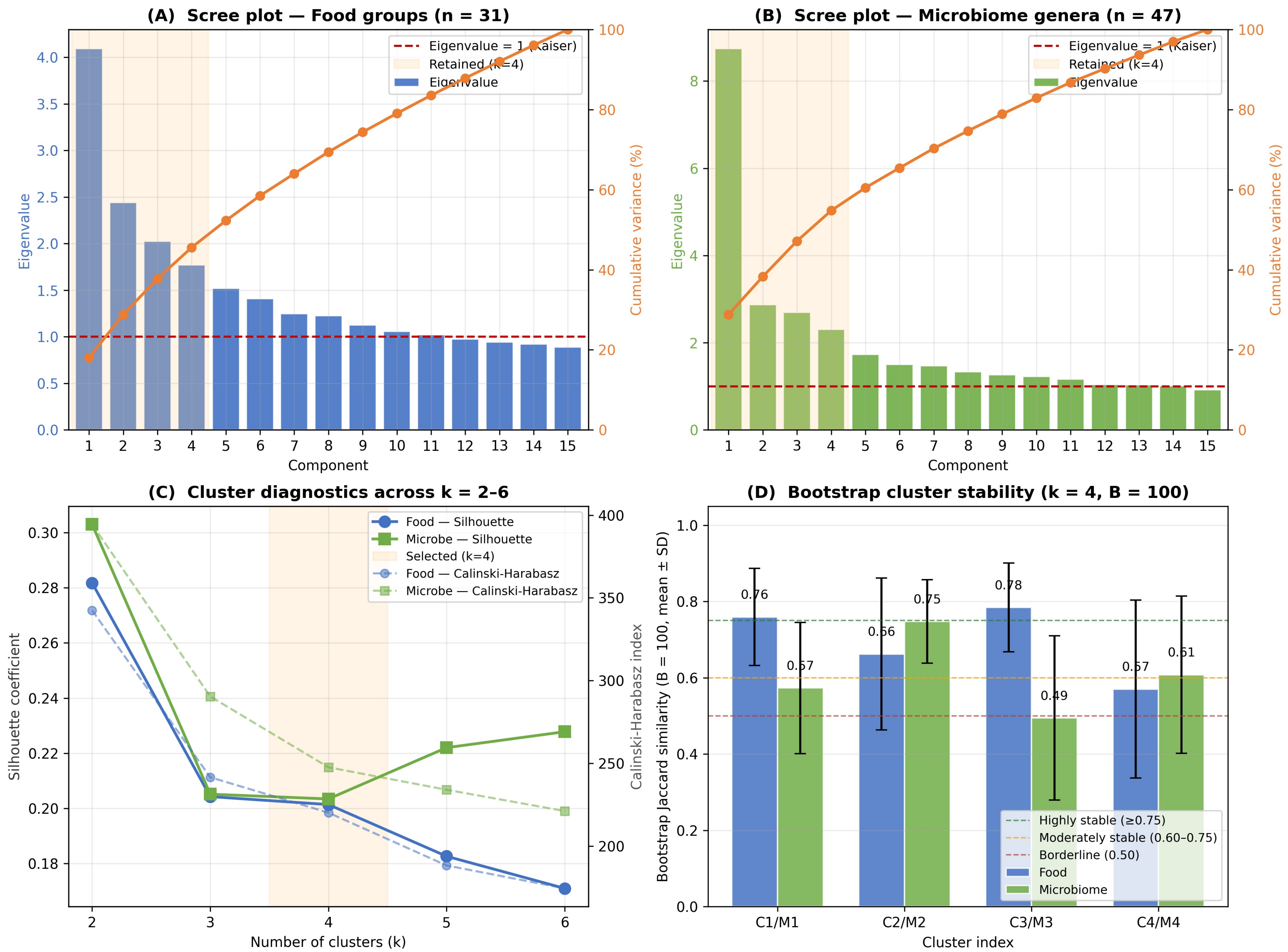

Supplement: Supplementary file 1 [file nutrients-18-02300-s001.zip › Figure S3_Cluster_diagnostics.pdf]
