# Supplementary material for: Joint Dietary and Gut Microbial Profiling and the Fatty Liver Index in Community-Dwelling Older Japanese: A Cross-Sectional, Hypothesis-Generating Analysis from the Kyotango Longevity Study
Source: Nutrients. 2026 Jul 14;18(14):2300. doi: 10.3390/nu18142300 (PMC13415844; doi:10.3390/nu18142300)
Supplement: Supplementary file 1 [file nutrients-18-02300-s001.zip › Supplementary Table S2.pdf]

Supplementary Table S2. NCBI sample registration list (SRA accession: PRJNA1470357, PRJNA1440982)

ID	Microbiota-ID	Age	Sex: M1, F2	FLI	FIB-4	Log[FLI]	Log[FIB4]	Log[FLI]_Z	Log[FIB4]_Z
1	99-0001945	75	1	56.28835205	1.697749375	#NUM!	#NUM!	1.178639835	-0.117001631
3	99-0001915	76	1	2.876376541	2.569412321	#VALUE!	#VALUE!	-2.068702388	1.052681951
4	99-0001916	68	1	42.00223555	1.316619586	1.750418534	0.229873579	0.858962159	-0.834652369
5	99-0001889	76	1	51.53876447	5.341201693	0.458845738	0.409833802	1.082382795	3.118313402
6	99-0001918	73	2	11.05823162	1.686275141	1.623272406	0.119460311	-0.59826615	-0.136144105
7	99-0001900	74	2	17.18401581	2.238698646	1.712134003	0.727638978	-0.116939902	0.663753115
8	99-0001914	68	2	10.34148124	1.489419088	1.043685682	0.226928438	-0.67143832	-0.486551664
9	99-0001952	79	1	8.390082249	3.294835752	1.235124663	0.349995637	-0.899774155	1.754647013
10	99-0001902	68	1	11.67911532	2.181101017	1.014582749	0.173016915	-0.53861732	0.590177689
11	0001-00321	69	2	5.394609051	1.78125	0.923766218	0.51783377	-1.382023788	0.018525101
12	0001-00323	71	1	12.33046206	1.595678398	1.067409947	0.33867578	-0.479357847	-0.292026361
13	0001-00322	67	2	6.315720911	1.859402979	0.731959977	0.250724877	-1.209890525	0.139735298
14	0001-00325	74	1	35.56455629	1.96144659	1.090979351	0.202945366	0.677294699	0.290547106
16	0001-00327	70	2	43.03206713	1.460518972	0.80042293	0.269373523	0.885411616	-0.541862059
18	0001-00329	68	2	58.48945513	1.041167044	1.551017395	0.292576487	1.220524899	-1.497228929
19	0001-00330	71	1	54.87955135	0.632307334	1.633792209	0.164507203	1.150962971	-2.905008544
20	0001-00331	67	2	5.016144778	3.118238994	1.767077576	0.017520413	-1.46144886	1.599146289
21	0001-00332	74	1	18.19934036	2.322372356	1.739410552	-0.199071781	-0.054257073	0.767333399
23	0001-00339	73	2	12.10044515	2.503966288	0.700370063	0.493909398	-0.499919427	0.979850943
24	99-0001917	76	1	64.64023585	3.567148954	1.260055647	0.365931853	1.329707281	1.978804509
25	0001-00283	79	2	2.147243729	3.008278146	1.082801348	0.398628477	-2.387923141	1.497807142

26	0001-00282	85	2	21.88988216	1.191588785	1.810502932	0.552321245	0.147355374	-1.116308813
27	0001-00284	78	2	16.21121147	2.026308981	0.331881343	0.478317989	-0.180573643	0.382382248
28	0001-00285	69	1	22.3442894	1.768735412	1.340243424	0.076126407	0.169790375	-0.001376956
29	0001-00286	76	2	19.06183009	1.407472295	1.209815471	0.306705669	-0.003698071	-0.646294655
30	0001-00287	77	2	46.76216741	1.763232594	1.349166548	0.247662871	0.976182283	-0.010172747
31	0001-00288	70	1	35.88665101	0.944653022	1.280164594	0.148439855	0.687139353	-1.771827961
32	0001-00289	72	1	13.17935605	1.529173514	1.669894632	0.246309605	-0.406658593	-0.412196306
33	0001-00290	84	2	46.07134776	2.217258883	1.554932931	-0.024727682	0.959930861	0.636589444
34	0001-00301	75	2	19.69146377	1.628824772	1.119894191	0.184456767	0.03178662	-0.233990732
35	0001-00302	82	2	21.36826428	1.774725488	1.663430917	0.345816404	0.12102063	0.008166624
36	0001-00303	77	2	30.62217354	1.12727168	1.294278001	0.211874366	0.513914993	-1.272936832
37	0001-00304	76	2	40.17371502	1.763048411	1.329769246	0.249131187	0.810360592	-0.010467623
38	0001-00343	72	2	61.64530251	3.02147848	1.486036013	0.052028597	1.277906243	1.510166389
39	0001-00344	82	1	2.211623043	2.066479603	1.603941995	0.246264238	-2.355665856	0.437794955
40	0001-00345	67	2	25.23631579	1.27468715	1.789899988	0.480219505	0.302692353	-0.926016422
41	0001-00346	73	1	51.42362836	1.390503013	0.344711106	0.315231123	1.079940734	-0.680534446
42	0001-00347	71	1	16.53692195	1.28520669	1.402025953	0.105403608	-0.158852485	-0.902816667
43	0001-00348	72	2	46.15011003	2.32056923	1.711162716	0.143171934	0.961795994	0.765140902
44	0001-00281	68	1	14.21182999	2.197757561	1.218454677	0.108972978	-0.324302155	0.611652644
46	0001-00308	68	2	35.97599815	1.417547874	1.664172741	0.365594529	0.689854548	-0.62615943
47	0001-00336	69	1	49.88928467	2.124765251	1.152650004	0.341979783	1.046864615	0.516309981
48	0001-00342	79	1	55.14803374	1.770049414	1.556012852	0.151537735	1.156291883	0.000719318
49	0001-00333	69	1	37.89004604	1.473840907	1.698007277	0.327310955	0.746456143	-0.516231204
50	0001-00334	75	2	35.35024941	1.214490858	1.741530033	0.247985391	0.670695002	-1.062570493

51	0001-00349	76	2	19.00156431	3.139698499	1.578525133	0.168450606	-0.007155769	1.618505889
52	0001-00338	71	1	6.754090567	2.752929269	1.548392482	0.08439425	-1.13661523	1.247420448
53	0001-00498	69	2	12.74787451	1.465250965	1.278789356	0.496887945	-0.443005683	-0.532731225
54	0001-00499	73	2	11.47009954	1.962660588	0.82956688	0.439795053	-0.55833601	0.292293665
55	0001-00500	86	1	24.96162444	2.96961326	1.10543778	0.165912016	0.290741831	1.461291331
56	0001-00306	76	2	39.45652535	1.350111399	1.059567187	0.292845202	0.790691187	-0.763745475
57	0001-00309	73	2	51.47139961	2.770673825	1.397272845	0.472699894	1.080954635	1.265556818
58	0001-00341	71	2	54.64188178	1.097940478	1.596118837	0.130369604	1.146223841	-1.34735685
59	0001-00335	74	1	15.75722681	2.696571757	1.711565978	0.442585402	-0.211588689	1.189033371
60	0001-00350	70	2	7.472343544	1.402671756	1.737525647	0.040578797	-1.026264741	-0.655938896
61	0001-00476	72	2	30.60980248	1.512131297	1.197479786	0.430811982	0.513473776	-0.443831964
62	0001-00305	80	2	3.954415035	1.859208639	0.873456831	0.146956052	-1.721140672	0.139440254
63	0001-00494	71	2	14.69543717	1.400477928	1.485860527	0.179589502	-0.28776374	-0.660357271
64	0001-00495	67	1	11.67235915	1.745743122	0.597082249	0.269328129	-0.539249163	-0.038311582
65	0001-00492	75	1	67.39118963	4.213250442	1.16718251	0.146276268	1.375215759	2.44870546
66	0001-00481	72	1	4.363991787	2.663820095	1.067158642	0.24198034	-1.613526382	1.154538988
67	0001-00491	68	1	36.53733362	1.281746032	1.828603123	0.624617275	0.706760389	-0.910427764
68	0001-00496	72	1	37.33397488	1.326806944	0.639883925	0.425504891	0.730312363	-0.812895187
69	0001-00483	72	1	14.47796349	1.303176493	1.562736851	0.107801982	-0.304043619	-0.863621917
70	0001-00482	66	2	9.843781615	0.736738032	1.572104231	0.122807736	-0.7252956	-2.473528233
71	0001-00488	70	2	33.32126765	1.608034888	1.160707477	0.115003237	0.606151667	-0.27025178
72	0001-00487	76	2	9.804122781	1.160771704	0.99316197	-0.132686911	-0.729703663	-1.190272516
73	0001-00486	75	2	9.055946831	1.911099409	1.522721515	0.206295467	-0.816382265	0.21714488
74	0001-00484	69	2	5.881787118	1.673299987	0.991408741	0.064746813	-1.287615243	-0.157948121

75	0001-00485	70	2	2.980738607	2.814941611	0.956933864	0.281283278	-2.029786374	1.310300532
76	0001-00307	69	1	22.33098137	1.226840622	0.769509302	0.223573808	0.169139841	-1.034011571
77	0001-00489	76	1	9.453317285	2.571120808	0.474323893	0.449469391	-0.769490495	1.054558284
78	0001-00497	72	2	32.10068616	1.409134177	1.348907809	0.088788148	0.565402649	-0.642963612
79	0001-00480	69	2	77.74623802	1.819057529	0.975584235	0.410122483	1.531291253	0.077812287
80	0001-00493	76	2	33.37132563	1.568056882	1.506514316	0.148952348	0.607790819	-0.341317016
81	0001-00478	68	1	79.00202711	0.844041192	1.890679384	0.259846434	1.548787574	-2.089718628
82	0001-00490	71	2	7.421587366	2.311124457	1.523373459	0.195361813	-1.033706999	0.753628699
83	0001-00472	73	2	38.48250488	1.674045991	1.897638235	-0.073636358	0.763397691	-0.15668993
84	0001-00474	70	1	36.18534549	1.559011538	0.870496804	0.363823333	0.696190145	-0.357647351
85	0001-00471	65	2	5.230584408	1.657573119	1.585263333	0.223767385	-1.415739337	-0.184604033
86	0001-00473	88	1	17.12236975	2.183767088	1.558532724	0.192849329	-0.120864131	0.593626004
87	0001-00475	67	2	31.66325874	1.556010929	0.718550215	0.219472695	0.550420948	-0.363085543
88	0001-00513	77	2	24.31141036	1.579101563	1.233563871	0.339206316	0.261921713	-0.321504368
89	0001-00514	76	2	5.738127415	1.980296732	1.50055561	0.192012643	-1.314616105	0.317545366
90	0001-00515	78	2	35.12451222	2.433866272	1.385810154	0.198410063	0.663699893	0.899698436
91	0001-00516	69	2	2.243728291	1.574404762	0.758770188	0.296730271	-2.339928743	-0.329912798
92	0001-00517	76	2	16.56149015	4.654030511	1.545610302	0.386296712	-0.15723146	2.729569247
93	0001-00519	72	1	23.20978693	1.679842102	0.350970264	0.197116395	0.211287149	-0.146933414
94	0001-00520	70	2	15.31228741	1.594757848	1.219099411	0.667829226	-0.242865292	-0.293655294
95	0001-00518	85	1	7.11248085	0.664860497	1.365671154	0.225268462	-1.080159589	-2.763300532
96	0001-00511	67	1	15.30517354	2.110284893	1.185040072	0.202694748	-0.243372703	0.497006811
97	0001-00512	69	1	6.606131307	2.479253056	0.85202111	-0.17726947	-1.160801545	0.951852797
98	0001-00479	92	1	1.753071515	3.934760995	1.184838258	0.32434109	-2.609382943	2.255672046

99	0001-00508	76	2	7.237748584	1.687639248	0.819947202	0.394320857	-1.061095552	-0.133861552
100	0001-00510	70	1	60.42033595	1.767269529	0.243799633	0.594918358	1.255989861	-0.00371737
101	0001-00509	65	2	16.9296426	1.405035001	0.859603493	0.227279617	-0.133224408	-0.651187039
102	0001-00501	70	1	11.37429495	1.29123847	1.781183136	0.247302789	-0.567494686	-0.889599707
103	0001-00502	69	2	15.93952674	2.477683386	1.22864779	0.147687143	-0.19902839	0.950065069
104	0001-00503	74	2	20.44233357	2.329082567	1.055924486	0.111006457	0.07264946	0.775477701
105	0001-00504	72	2	36.53123952	1.435514919	1.202475423	0.394045809	0.706578251	-0.590606349
106	0001-00506	73	2	32.52233023	1.892592593	1.310530471	0.367184885	0.579651781	0.189676315
107	0001-00505	66	1	37.96265309	1.674211192	1.562664408	0.15700771	0.748546553	-0.156411381
108	0001-00638	78	1	57.86960171	3.098557805	1.512181655	0.277057136	1.208891238	1.581273502
109	0001-00639	69	2	41.16441862	1.600515464	1.579356556	0.223810241	0.836961379	-0.283482476
110	0001-00507	68	1	12.27233845	1.317968065	1.762450493	0.491159603	-0.484517165	-0.831762769
111	0001-00640	71	2	5.740554136	0.92431037	1.614521986	0.204259875	-1.314154414	-1.833279124
112	0001-00637	87	2	24.62558103	1.793978085	1.088927324	0.119904887	0.27594203	0.038623771
113	0001-00636	69	2	4.104857096	0.816335422	0.758953817	-0.034182175	-1.680370045	-2.183931472
114	0001-00635	71	2	70.13336883	1.434343434	1.391386486	0.253817133	1.418766607	-0.592910879
115	0001-00634	69	2	29.29872522	1.108044462	0.613298042	-0.088131359	0.465673273	-1.321498588
116	0001-00632	69	2	17.63289205	2.017384996	1.845924701	0.15665315	-0.088783059	0.369923127
117	0001-00631	72	2	82.83987708	1.126617097	1.466848725	0.044557188	1.600584253	-1.274576435
118	0001-00670	69	2	4.528460898	1.587332206	1.246323549	0.304788787	-1.573130621	-0.306829624
119	0001-00677	70	2	15.50314308	2.702964627	1.918239446	0.051776338	-0.229339402	1.195717514
120	0001-00678	70	2	3.982808131	1.714198257	0.655950622	0.200667828	-1.713328541	-0.089784423
121	0001-00679	73	1	14.98108745	2.42246085	1.190419755	0.431840362	-0.266742492	0.886439446
122	0001-00680	74	1	17.8547532	0.998247658	0.600189385	0.234061049	-0.075129898	-1.616056786

123	0001-00671	69	2	14.0103855	1.200437442	1.175543339	0.384256767	-0.339890326	-1.095424529
124	0001-00672	70	2	53.75510206	1.212435565	1.251753851	-0.0007617	1.128357661	-1.067351543
125	0001-00674	71	2	16.01398753	2.024167266	1.146450085	0.079339533	-0.193939381	0.379397129
126	0001-00675	75	1	49.7018202	1.275775908	1.730419691	0.083658667	1.042753853	-0.923606415
127	0001-00673	75	1	53.52341964	1.021858884	1.204499486	0.306246397	1.123641328	-1.550067988
128	0001-00669	75	1	48.67158888	1.643128556	1.696372294	0.105774396	1.019882262	-0.209310298
129	0001-00668	88	2	11.24062832	2.403402926	1.728543853	0.009390925	-0.580402611	0.86414437
130	0001-00667	73	2	12.29230012	1.908863715	1.687275524	0.215671543	-0.482742527	0.213840734
131	0001-00666	74	1	37.52469445	2.587713344	1.050790588	0.380826586	0.735876241	1.07271632
132	0001-00665	76	2	11.16969343	1.374691791	1.089633155	0.280774923	-0.587315136	-0.712815716
134	0001-00664	68	2	15.75541081	1.418876916	1.574317165	0.412916165	-0.21171454	-0.623514139
135	0001-00663	82	1	44.91212051	1.650218167	1.048041253	0.138205339	0.932104714	-0.197157065
136	0001-00661	77	2	20.01157612	2.70601561	1.197429731	0.151944723	0.049394682	1.198901936
137	0001-00662	68	1	26.4059377	0.745146929	1.652363561	0.217541364	0.352161915	-2.441492447
139	0001-00652	84	1	29.770422	1.761519329	1.301281295	0.432330297	0.483112809	-0.012916861
140	0001-00653	84	2	16.57694227	1.346283213	1.421701594	-0.127758084	-0.156213151	-0.771760706
141	0001-00654	69	2	7.747482288	1.3796515	1.473784991	0.245887413	-0.986781494	-0.702649839
142	0001-00655	77	2	7.871103871	2.805134716	1.219504425	0.129136431	-0.969495867	1.300449178
143	0001-00657	72	2	29.56030596	1.643478261	0.889160592	0.139769398	0.475378798	-0.208709595
144	0001-00658	74	1	10.54814518	1.273355014	0.896035644	0.447953723	-0.649832454	-0.928967959
145	0001-00659	74	2	3.904286545	1.203490702	1.470708925	0.215763964	-1.735071059	-1.088254041
146	0041-00094	90	2		2.677904394	1.023176099	0.104949503		1.169424402
147	0041-00095	91	2		2.828427125	0.591541684	0.080442739		1.323791282
148	0001-00660	72	2	16.85081022	2.007704544	#NUM!	0.427795068	-0.138320807	0.356345398

149	0041-00092	76	2	27.23812584	2.864419306	#NUM!	0.451544993	0.386043067	1.359484907
151	0041-00096	80	1	10.30541471	3.465461578	1.226620787	0.302699802	−0.675253135	1.89716753
152	0001-00656	80	2	17.19182677	2.432360427	1.435177222	0.457036592	−0.116443681	0.89795143
153	0041-00097	87	1	13.64500742	1.351465653	1.013065473	0.539761088	−0.368744683	−0.760915459
154	0041-00099	70	2	4.391676718	1.626238114	1.235322027	0.386027929	−1.606621137	−0.238477008
155	0041-00051	70	2	11.38942525	1.52173913	1.134973776	0.130805013	−0.566043151	−0.425953258
156	0041-00052	70	1	82.83712364	1.732084219	0.642630363	0.211184135	1.600547959	−0.060484162
157	0041-00053	65	2	9.428411365	2.125831392	1.056501809	0.182340208	−0.772371105	0.517726005
158	0041-00054	68	2	45.54197775	1.440386245	1.91822501	0.238569005	0.947311743	−0.581043665
159	0041-00055	72	2	13.46392348	0.982748197	0.974438523	0.327528816	−0.383332741	−1.660228887
160	0041-00057	65	2	2.162888961	1.622449323	1.658411887	0.158478965	−2.379995997	−0.245061147
161	0041-00093	87	2	61.40589286	1.780537733	1.129171635	−0.007557744	1.273657311	0.017396135
162	0041-00058	74	1	14.35602589	2.073529253	0.335034224	0.210171141	−0.313279088	0.447408264
163	0041-00059	70	1	30.6350634	2.775647151	1.78821005	0.250551182	0.514374523	1.270619121
164	0041-00060	69	2	66.89103325	2.204605262	1.157034233	0.316710167	1.367081598	0.620434079
165	0041-00056	67	1	30.29074166	1.191111111	1.486218783	0.443364256	0.502032361	−1.117440609
166	0041-00066	74	2	48.30140134	1.372917415	1.825367904	0.34333084	1.011545515	−0.71646155
167	0041-00065	70	2	27.81964514	2.175802581	1.481309907	0.075952276	0.40910975	0.583312127
168	0041-00064	78	1	18.61351468	1.99751746	1.683959731	0.137644414	−0.029685924	0.341986192
169	0041-00063	68	2	29.90041964	2.119539285	1.444351586	0.337619488	0.487870514	0.509358675
170	0041-00062	68	2	10.26763775	1.207779368	1.269828386	0.300490584	−0.679263208	−1.078212901
171	0041-00061	69	2	87.89291232	1.30442992	1.475677283	0.326241471	1.665236813	−0.860908208
173	0041-00077	68	2	4.391830798	1.592546369	1.011470538	0.081987606	−1.606582828	−0.297572402
174	0041-00078	75	2	56.30126456	2.745023085	1.943953855	0.115420752	1.178890293	1.239302012

175	0041-00079	71	2	3.12172202	2.794873878	0.6426456	0.202092086	-1.979324451	1.290104886
176	0041-00080	67	2	4.540730929	1.907178218	1.75051815	0.438546001	-1.570176007	0.211347166
177	0041-00068	75	2	9.012032352	1.62553283	0.494394228	0.446362215	-0.821690162	-0.239701483
178	0041-00091	77	1	32.77302395	2.900684932	0.657125768	0.280391278	0.588036466	1.394998934
179	0041-00069	73	2	10.65711648	1.626850342	0.954822742	0.210995745	-0.638609794	-0.237414521
180	0041-00070	75	1	7.514649178	1.41512853	1.515516515	0.462500559	-1.020100092	-0.630981204
181	0041-00089	68	2	61.59286626	1.115203747	1.027639713	0.211347603	1.276977042	-1.303318777
182	0041-00090	70	2	45.94937742	2.30869284	0.87590871	0.150795887	0.957036235	0.750657193
183	0041-00074	66	2	17.54191109	1.468014251	1.789530415	0.04735422	-0.094431691	-0.527412827
184	0041-00075	70	2	80.59321837	1.195121951	1.662279631	0.363366156	1.570561679	-1.107951421
185	0041-00073	67	2	70.6637625	0.982411767	1.244076905	0.166730272	1.426993391	-1.661195391
186	0041-00072	70	2	25.72707388	1.770658858	1.906298499	0.077412223	0.323722682	0.001691057
187	0041-00071	77	2	14.24168928	2.29345091	1.849196758	-0.007706444	-0.322010406	0.731959521
188	0041-00085	68	2	9.738543426	2.597943667	1.410390394	0.248134896	-0.73703205	1.083853937
189	0041-00086	79	2	23.03456061	2.850666954	1.153561506	0.360489449	0.203012182	1.345899859
190	0041-00084	69	1		1.400279764	0.988494005	0.41462973		-0.660756714
191	0041-00087	66	2	16.52901782	1.306930693	1.362379932	0.454946481	-0.159374517	-0.855501738
192	0041-00088	69	1	35.23005353	1.131122394	#NUM!	0.146214813	0.666975969	-1.263310782
193	0041-00139	71	1	53.80041018	1.780853042	1.218247048	0.116252557	1.129277616	0.017895966
194	0041-00083	74	2	4.023228259	2.610906217	1.546913303	0.053509601	-1.702302813	1.09790327
195	0041-00140	68	2	12.15231479	2.094535996	1.730785587	0.250628083	-0.495248788	0.475861657
196	0041-00143	77	1	16.13572837	3.041381265	0.604574673	0.416791272	-0.185669777	1.528699318
197	0041-00150	83	1	10.39386408	2.504496477	1.084659011	0.321087828	-0.665921331	0.980448573
198	0041-00148	66	2	8.967975844	2.332966394	1.207788574	0.483070866	-0.82704128	0.780180856

199	0041-00161	69	1	51.08959844	1.312545735	1.016777033	0.398720425	1.072824823	−0.843400066
200	0041-00158	71	2	6.084024028	1.603757463	0.95269443	0.367908483	−1.250701923	−0.277770455
201	0041-00146	66	2	7.902387183	1.478940061	1.708332489	0.118114445	−0.96516466	−0.506481902
202	0041-00145	70	1	16.95560188	1.319494333	0.784190921	0.20513869	−0.131551371	−0.828495757
203	0041-00157	69	2	17.60129093	2.427777778	0.897758304	0.169950573	−0.090741732	0.892628212
204	0041-00159	69	1	13.82829443	1.788059047	1.229313211	0.120407529	−0.354174978	0.029294931
205	0041-00155	75	2	2.663982588	1.685803444	1.245544521	0.385208932	−2.152463145	−0.136933822
206	0041-00132	68	1	52.89880615	1.769924706	1.140768618	0.252381856	1.110823705	0.000520434
207	0041-00160	77	1	9.842589742	3.060018299	0.425531382	0.226806937	−0.725427817	1.545943987
208	0041-00149	69	1	20.75198507	1.950700641	1.723445871	0.247954792	0.089065466	0.275039781
209	0041-00147	75	1	23.38908757	1.49508262	0.993109383	0.485724024	0.219690103	−0.47583839
210	0041-00144	74	1	52.45354567	1.517401588	1.317059646	0.290190627	1.101593826	−0.434010741
211	0041-00152	76	2	9.617334811	1.771898416	1.36901328	0.174665193	−0.750707757	0.003666463
214	0041-00067	69	2	31.44394683	1.810776541	1.71977485	0.181100534	0.54283153	0.064932689
216	0041-00135	70	1	26.85893889	1.49921936	0.983054736	0.24843882	0.370735356	−0.468038849
217	0041-00156	75	1	68.94291446	2.243283763	1.497537053	0.257864859	1.400072972	0.669528575
218	0041-00154	76	2	1.596812009	2.08369737	1.429088851	0.175865182	−2.711325496	0.461216681
219	0041-00136	71	1	24.10755792	2.264556145	1.838489639	0.350884213	0.252727249	0.696169975
220	0041-00153	77	2	11.8507923	1.728483243	0.20325379	0.318834644	−0.522683385	−0.066358774
221	0041-00175	79	1	28.78224013	3.248099485	1.382153219	0.354983093	0.446252841	1.714320101
222	0041-00180	76	2	69.92715051	1.52349866	1.073747387	0.237665174	1.415551205	−0.422691276
223	0041-00177	65	1	55.57873921	1.033736714	1.459124592	0.511629323	1.164786688	−1.517445999
224	0041-00173	65	1	13.09839165	1.14199186	1.844645831	0.182842077	−0.413387287	−1.236314968
225	0041-00134	71	1	70.16493006	2.692527668	1.744908691	0.014409941	1.419257883	1.184796836

226	0041-00141	76	2	37.14547693	1.185611181	1.117217972	0.057663008	0.724785299	-1.130504896
227	0041-00169	78	1	27.1972898	3.112074303	1.846120097	0.430160175	0.384404797	1.593560202
228	0041-00163	76	1	78.63401844	2.289961674	1.569905939	0.073942286	1.543689258	0.727661705
229	0041-00166	69	1	60.19621873	1.715908323	1.434525629	0.493049958	1.251932046	-0.086969857
230	0041-00131	65	2	21.3227836	1.025960601	1.89561047	0.359828214	0.118694073	-1.538760117
231	0041-00142	71	2	55.17739765	1.143925301	1.779569212	0.234494081	1.156873132	-1.231539931
232	0041-00179	69	2	21.50856124	1.265659789	1.328843899	0.011130683	0.128166427	-0.946078495
233	0041-00133	74	1	33.74400704	2.490423445	1.741761214	0.058397666	0.619917555	0.964542373
234	0041-00176	66	1	46.31022592	1.817816634	1.33261136	0.102316982	0.965577831	0.075886036
236	0041-00162	68	1	16.1537276	2.818673928	1.528196653	0.396273196	-0.184452423	1.314040756
237	0041-00171	67	2	17.09312663	1.587094093	1.6656769	0.259550073	-0.122730615	-0.307253096
238	0041-00172	71	2	71.93576604	1.684072849	1.208272755	0.450044838	1.44647412	-0.139833086
239	0041-00165	74	2	5.877530865	1.846059695	1.23282151	0.200602675	-1.288405683	0.11940572
240	0041-00164	75	2	19.31935306	3.210030331	1.856944873	0.226360874	0.010954965	1.681040534
241	0041-00167	74	1	8.858181871	1.067307692	0.769194918	0.26624574	-0.84049213	-1.42723242
242	0041-00138	68	2	30.14042404	1.474885845	1.285992579	0.506509136	0.496600191	-0.514230596
243	0041-00174	79	2	2.417779462	1.775913486	0.947344593	0.028289639	-2.258350198	0.010055551
244	0041-00168	69	1	79.17939583	4.755700685	1.479149358	0.168758407	1.551236328	2.790570594
245	0041-00178	74	2	2.551880856	1.664225001	0.383416684	0.249421805	-2.199406754	-0.173298839
246	0041-00196	68	2	6.779927732	1.75575245	1.898612184	0.677214513	-1.132446132	-0.022173247
247	0041-00186	83	2	28.22554486	2.14392193	0.406860394	0.221212042	0.424926289	0.541645802
248	0041-00187	83	2	51.89762132	2.014852314	0.831225065	0.244463283	1.089959373	0.36637711
249	0041-00195	75	2	48.75024713	1.856268386	1.450642334	0.331208967	1.021645502	0.134972632
250	0041-00190	72	2	42.9047949	0.90773754	1.715147453	0.304243219	0.882177333	-1.884350537

251	0041-00191	80	2	8.198516801	1.308886457	1.687976822	0.268640768	-0.924994456	-0.851280738
252	0041-00192	69	2	4.282893463	1.835095972	1.63250583	-0.042039704	-1.634009138	0.102591322
253	0041-00181	75	1	24.02424857	2.972560976	0.913735291	0.116901974	0.248947304	1.464091901
254	0041-00185	75	1	55.43815188	1.073731688	0.631737271	0.263658782	1.162021143	-1.410293408
255	0041-00197	69	2	16.36251345	1.687428418	1.380649813	0.473130772	-0.170429771	-0.134214213
256	0041-00198	78	2	48.64798363	1.421045512	1.743808744	0.03089577	1.019352559	-0.619203134
257	0041-00200	89	2	4.674330699	3.630263158	1.213850016	0.227225359	-1.538512328	2.028311673
258	0041-00199	72	1	3.788375436	2.034228182	1.687064844	0.152607987	-1.767979307	0.393392687
259	0041-00189	67	1	32.52777731	1.429559176	0.669719435	0.559938108	0.57983465	-0.602341992
260	0041-00193	79	1	15.18760714	1.546745956	0.578453012	0.308399667	-0.251792698	-0.379943459
261	0041-00194	72	2	44.59778892	2.016614871	1.512254388	0.155202138	0.924435653	0.368845343
262	0041-00183	68	2	8.071708011	1.708133971	1.181489355	0.189418989	-0.942015573	-0.099788203
263	0041-00182	71	2	5.334915904	1.504237288	1.649313328	0.304622965	-1.394173667	-0.458606721
264	0041-00117	71	1	26.78573931	1.200198396	0.906965443	0.23252193	0.367755428	-1.09598669
265	0041-00119	72	1	48.4387707	1.452603558	0.727127578	0.17731635	1.014646551	-0.557201945
266	0041-00118	71	2	6.77306048	1.790168815	1.427903638	0.079253042	-1.133552683	0.032623619
267	0041-00126	73	2	9.450684371	1.552230972	1.685193113	0.162147104	-0.769794658	-0.369951141
268	0041-00120	72	1	49.69784635	1.376494403	0.830784954	0.252893987	1.042666546	-0.709116679
269	0041-00121	78	2	25.93060578	1.558984192	0.975463259	0.190956345	0.332327136	-0.357696865
270	0041-00122	69	2	23.97684798	1.501242089	1.696337569	0.13877445	0.24679077	-0.464232961
271	0041-00123	68	2	34.33215877	1.418206355	1.413812663	0.192841711	0.638785673	-0.624848496
272	0041-00124	73	2	44.40553651	1.804645432	1.37979209	0.176450732	0.919718394	0.055358838
273	0041-00125	69	2	12.11187493	2.042763158	1.535701112	0.151739427	-0.498888506	0.405211376
274	0041-00127	83	1	16.09121333	2.55314005	1.647437122	0.256391886	-0.188686333	1.034748273

275	0041-00129	82	2	45.53244086	2.673962051	1.083211378	0.310218017	0.94708306	1.165265723
276	0041-00205	79	1	56.38472549	1.438080614	1.206588792	0.407074638	1.180507765	-0.585565708
277	0041-00128	65	2	2.535333396	1.647853549	1.658320932	0.427155239	-2.206510318	-0.201204756
278	0041-00188	74	2	11.08562348	1.730882248	1.75116147	0.157783232	-0.59556473	-0.062443691
279	0041-00203	79	1	25.51812052	2.581699346	0.404035077	0.216918612	0.314817925	1.066148391
280	0041-00201	78	2	20.71507953	1.6	1.044760124	0.238267524	0.087121844	-0.284391728
281	0041-00206	67	1	83.17796706	1.404598997	1.406848684	0.411905665	1.605031606	-0.652063126
282	0041-00207	68	2	44.9776887	1.478698569	1.316286605	0.204119983	0.933697679	-0.506942864
283	0041-00208	69	2	17.98786094	1.426691729	1.920008302	0.147552354	-0.067019743	-0.608009672
284	0041-00290	74	1	35.86946206	1.691414366	1.652997134	0.169879653	0.686616218	-0.127554277
285	0041-00209	82	2	28.20301445	2.05469665	1.254979522	0.154330144	0.424054335	0.421653588
286	0041-00210	88	1	33.04891482	4.210741133	1.554724864	0.228250015	0.597190082	2.447023783
287	0041-00211	71	1	64.33715865	1.489360461	1.45029553	0.312747713	1.324575549	-0.486662777
288	0041-00212	72	1	5.620168197	3.598055327	1.519157204	0.624358543	-1.337296876	2.003156156
289	0041-00213	75	1	4.796717192	2.102803738	1.808461877	0.17299982	-1.510290632	0.486982036
290	0041-00215	75	1	70.1785079	2.344396859	0.749749313	0.556067837	1.419469164	0.793977385
291	0041-00216	74	2	3.612146321	1.567347948	0.680944114	0.32279874	-1.819993385	-0.342593509
292	0041-00214	71	2	30.7856638	1.537971572	1.84620413	0.370031131	0.519729228	-0.396002092
293	0041-00217	73	2	17.49229659	2.121320344	0.557765334	0.19516542	-0.097524407	0.511729668
294	0041-00269	69	2	23.00740617	1.586678631	1.488348522	0.186948308	0.201724198	-0.307992125
295	0041-00268	70	1	86.12378757	1.547086945	1.242846832	0.326606257	1.643034103	-0.37932123
296	0041-00264	78	2	5.774819521	2.389515892	1.361867659	0.200488973	-1.307656076	0.847786874
297	0041-00266	81	2	15.0028833	2.968586387	1.935123121	0.189514721	-0.265155012	1.460315066
298	0041-00289	70	2	41.09488834	1.170297876	0.761538416	0.378309923	0.835115461	-1.167201232

299	0041-00288	68	1	32.04396653	1.985057222	1.176174731	0.472549692	0.563471586	0.324322967
300	0041-00265	89	1	53.30938052	3.265791666	1.613787805	0.068296417	1.11926598	1.729653842
301	0041-00263	98	1	4.176709193	2.552972008	1.505746269	0.29777303	-1.661422134	1.034562477
302	0041-00270	68	2	17.90616242	1.065521679	1.726803636	0.513988476	-0.071990429	-1.431959957
303	0041-00262	73	2	26.86157644	1.478954856	0.620834238	0.407046053	0.370842578	-0.506453664
304	0041-00267	74	1	41.19181646	0.661228704	1.253002519	0.02756229	0.837687892	-2.778762176
305	0041-00256	82	1	11.07601579	2.001362538	1.429131497	0.169954918	-0.596511492	0.347414609
306	0041-00257	72	1	10.91752441	1.645986372	1.614810944	-0.179648302	-0.6122492	-0.204405044
307	0041-00258	69	1	9.845794322	1.532002986	1.044383566	0.301325766	-0.725072362	-0.406978074
308	0041-00259	76	2	30.89725226	1.942184758	1.038124172	0.216426235	0.523679967	0.262689838
309	0041-00261	77	2	92.18745122	2.408407931	0.993250759	0.185259612	1.717327011	0.87001659
310	0041-00260	77	2	29.40943489	1.826318213	1.489919859	0.288290541	0.469791508	0.089056835
311	0041-00287	101	1	7.268058829	4.481856232	1.964671808	0.381730049	-1.056532329	2.623161029
312	0041-00286	67	1	28.57857746	1.49767297	1.46848668	0.26157645	0.43849892	-0.470951939
313	0041-00255	75	1	32.79176061	1.355672966	0.861418434	0.651457921	0.588660554	-0.752141392
314	0041-00204	76	2	12.09950989	0.57862844	1.456040607	0.175416992	-0.500003826	-3.155431037
315	0041-00245	85	1	30.90925568	3	1.515764735	0.132154936	0.524104093	1.490028756
316	0041-00249	76	2	16.38690863	3.577708764	1.082767779	-0.237600224	-0.168803009	1.987148408
317	0041-00248	70	2	3.309209869	2.308656697	1.490088547	0.477121255	-1.915638186	0.750613001
318	0041-00247	75	1	15.88791963	1.978060134	1.214497032	0.553604985	-0.202569436	0.314355449
319	0041-00253	77	2	7.999608443	2.053575493	0.519724311	0.363359357	-0.9518129	0.420112904
320	0041-00250	74	2	13.57208753	1.982037522	1.201067034	0.29623949	-0.374595664	0.32002565
321	0041-00252	69	1	20.53044565	2.547304751	0.90306873	0.312510673	0.077345849	1.028289329
322	0041-00251	67	2	9.112852494	1.528603932	1.132646652	0.297111872	-0.809542296	-0.41324792

323	0041-00246	75	2	12.32456761	1.197893559	1.312398377	0.406080906	-0.479879956	-1.101412701
324	0041-00254	76	1	11.70314031	1.792222446	0.959654341	0.184294972	-0.536373435	0.03585997
325	0041-00275	73	2	12.50906299	2.096570159	1.090771692	0.07841823	-0.463655253	0.478601738
326	0041-00273	76	1	82.76773896	1.932457574	1.068302412	0.253391912	1.599632974	0.248516812
327	0041-00274	72	2	50.78444108	1.493087558	1.097224779	0.3215094	1.066283206	-0.479607661
328	0041-00276	68	2	44.78530506	1.690893695	1.917861091	0.286109968	0.92901715	-0.128423351
329	0041-00277	70	1	41.27431348	1.796431875	1.705730677	0.174085276	0.839872564	0.042482104
330	0041-00242	74	1	9.498649694	2.235296694	1.651135537	0.228116305	-0.764266788	0.659460332
331	0041-00243	74	2	64.84586324	1.091163139	1.615679858	0.254410752	1.333175301	-1.3648352
332	0041-00244	79	2	5.700321306	1.677606178	0.977661871	0.349335176	-1.321834156	-0.150693121
333	0041-00285	77	2	26.37578847	2.173687799	1.811882276	0.037889687	0.350914485	0.580567183
334	0041-00202	69	2	12.97449236	2.765485082	0.755899336	0.224690017	-0.423765115	1.260265548
335	0041-00284	89	1	17.01895457	3.081077254	1.421205451	0.337197168	-0.127479113	1.565303697
336	0041-00271	67	2	68.43026992	0.526401715	1.113090375	0.44177132	1.391923305	-3.422453948
337	0041-00272	80	1	31.8540346	1.900741627	1.230932879	0.488702588	0.556980229	0.20180439
338	0041-00281	80	1	4.772175606	2.266516931	1.835248253	-0.278682705	-1.515891629	0.698613042
339	0041-00280	77	2	14.32546354	2.68159204	1.503164448	0.278923086	-0.315606155	1.173308869
340	0041-00282	72	1	37.75127782	1.482454218	0.678716416	0.355358968	0.742449738	-0.499782574
341	0041-00241	68	2	34.0119313	2.274702699	1.156108684	0.428392708	0.628553113	0.708789428
342	0041-00279	70	1	43.18383752	2.104056166	1.576931656	0.17098129	0.889255972	0.488662777
343	0041-00278	81	2	5.535517671	1.281990535	1.531631293	0.356924643	-1.353868505	-0.909889349
344	0041-00240	71	2	37.55308661	1.619863869	1.635321233	0.323057329	0.736702109	-0.249562968
345	0041-00237	88	1	32.11357347	3.335091863	0.743158241	0.107884819	0.565840932	1.788926518
346	0041-00232	65	2	74.09779957	1.109437751	1.574645639	0.209478519	1.478808531	-1.317951376

347	0041-00238	87	1	45.67726463	2.683281573	1.506688635	0.523107801	0.950550609	1.175086794
348	0041-00233	73	1	12.74229909	2.788064049	1.869805311	0.04510294	-0.443483354	1.283218683
349	0041-00234	65	2	3.673736623	1.579270975	1.659700088	0.428666248	-1.801532014	-0.321201546
350	0041-00236	74	2	4.650888359	1.501711195	1.105247795	0.445302746	-1.544002252	-0.463351043
351	0041-00345	76	2	53.39882422	2.350073727	0.565108018	0.198456654	1.121096504	0.800804365
352	0041-00235	66	1	4.30407457	1.49078804	0.667535915	0.176586418	-1.628622309	-0.483958393
353	0041-00231	75	2	3.725326567	2.25782453	1.727531694	0.371081487	-1.786304856	0.687766506
354	0041-00343	70	1	11.86946398	0.839387098	0.633879787	0.1734159	-0.520964339	-2.105326648
355	0041-00239	73	2	24.37871922	1.780229966	0.57116435	0.353690187	0.264940657	0.016908175
356	0041-00346	69	1	28.72459757	2.973415544	1.074431107	-0.07603771	0.444063833	1.464903292
357	0041-00344	77	1	16.519001	1.545074072	1.387010885	0.250476107	-0.16003644	-0.382996258
358	0041-00347	74	2	11.15076108	1.573711333	1.458253953	0.473255608	-0.589167496	-0.331156332
359	0041-00338	78	2	1.923092247	2.016895291	1.217983779	0.188949305	-2.508308711	0.369237837
360	0041-00340	68	2	55.44547252	1.476834978	1.04730451	0.196925072	1.162165323	-0.510502626
361	0041-00351	73	2	38.57311156	1.054913295	0.284000117	0.304683352	0.765965599	-1.460204469
362	0041-00337	70	2	17.90970294	1.239460844	1.743866089	0.16933197	-0.071774548	-1.005122685
363	0041-00339	73	1	29.07223983	1.291156463	1.586284673	0.023216766	0.457199654	-0.889778989
364	0041-00350	70	1	22.81555993	1.021756008	1.253088382	0.093232811	0.192581042	-1.550352186
365	0041-00352	83	2	21.08926711	2.351087942	1.463478493	0.110978873	0.106669874	0.802022319
366	0041-00348	73	1	4.369258882	2.015060192	1.358231132	0.0093472	-1.612209282	0.366668327
367	0041-00342	75	2	8.137684235	2.706329387	1.324061487	0.371268874	-0.933126702	1.199229233
368	0041-00341	75	2	6.566059876	1.638683813	0.640407778	0.304288023	-1.167445117	-0.216956387
369	0041-00336	82	1	57.84356314	2.327501208	0.910500834	0.432380653	1.208399812	0.773560493
370	0041-00335	71	2	10.46332552	3.375	0.817304839	0.214495164	-0.658648339	1.822503703

371	0041-00334	81	1	46.76533475	2.398477775	1.762255037	0.366889915	0.97625624	0.858353891
372	0041-00324	81	1	18.9336293	3.741229744	1.019669737	0.528273777	-0.011066657	2.113303225
373	0041-00331	83	1	39.01562824	2.435984989	1.669924047	0.379935699	0.778421041	0.902154633
374	0041-00330	77	2	53.82949373	2.775787689	1.27723387	0.573014379	1.129867733	1.270762042
375	0041-00329	69	1	31.01181519	1.482844391	1.591238604	0.386674608	0.527721199	-0.499039734
376	0041-00327	83	2	11.02226352	1.87918842	1.731020295	0.443386245	-0.601823547	0.169613053
377	0041-00326	76	2	9.441535766	1.409754874	1.491527187	0.171095579	-0.770852194	-0.641720506
378	0041-00328	66	2	10.53228918	1.591812475	1.04227079	0.273970328	-0.651475076	-0.298873524
379	0041-00325	72	2	8.140567043	1.540814559	0.975042643	0.149143605	-0.932739951	-0.390788925
380	0041-00364	67	1	41.76108705	1.576470588	1.022522775	0.201891904	0.852674987	-0.326211374
381	0041-00363	93	1	71.68875875	4.696193274	0.910654657	0.187750373	1.442718298	2.755026772
382	0041-00349	71	2	33.51485025	1.468374598	1.620771795	0.197685873	0.612476947	-0.526720018
383	0041-00362	72	1	36.80036345	1.730769231	1.855451061	0.671745962	0.714592937	-0.062628009
384	0041-00361	72	1	53.23632219	1.756445889	1.525237283	0.166836863	1.117768512	-0.021058605
385	0041-00365	82	1	22.05079449	2.309378101	1.565852108	0.238239166	0.155352759	0.751494918
386	0041-00373	69	2	31.58904429	1.781572339	1.726208045	0.244634775	0.547858614	0.019035871
387	0041-00374	84	1	2.897282384	2.461845715	1.343424242	0.363495043	-2.060794838	0.931963617
388	0041-00323	78	2	24.59942973	3.265095172	1.499536487	0.250803461	0.274781834	1.729051766
389	0041-00375	80	1	13.07585535	2.662970661	0.461990826	0.391260832	-0.415267608	1.153638723
390	0041-00376	76	1	60.94703855	2.003806525	1.390925039	0.513895845	1.265467272	0.350859569
391	0041-00377	73	1	86.70546806	1.821599493	1.116470107	0.425366382	1.650384188	0.0817541
392	0041-00378	73	2	5.42522677	1.902997052	1.784952608	0.301855786	-1.375843955	0.205151916
393	0041-00379	77	1	9.465414167	3.132327501	1.938046487	0.260452897	-0.76809411	1.611871135
394	0041-00380	73	1	55.14439452	1.750888776	0.734417896	0.279438115	1.156219825	-0.030003569

395	0041-00372	69	1	60.36582767	1.175603864	0.976139622	0.495867164	1.255004333	-1.154432042
396	0041-00371	66	1	24.91641726	1.491649117	1.741501373	0.243258559	0.288762485	-0.482328433
397	0041-00384	75	1	14.96630514	2.788461538	1.78079116	0.070261005	-0.267820462	1.283621092
398	0041-00385	75	1	76.93821102	1.503870697	1.396485595	0.173666675	1.519883329	-0.459294732
399	0041-00386	70	2	2.199365364	1.484371038	1.175114596	0.445364659	-2.361734574	-0.496135071
400	0041-00387	82	2	28.27215126	3.148876265	1.886142084	0.177210497	0.426727807	1.626745206
401	0041-00388	85	2	13.19208554	2.860685145	0.342297381	0.171542472	-0.405604446	1.355802635
402	0041-00389	71	2	70.69439825	1.329487562	1.451358856	0.498155595	1.427466685	-0.807197939
403	0041-00390	68	1	54.5594755	0.929790365	1.120313459	0.456470061	1.144575846	-1.816593045
404	0041-00370	81	1	34.31715151	2.13014084	1.849385002	0.123684279	0.638308265	0.523442493
406	0041-00382	65	1	58.9352646	1.160678303	1.736870187	-0.031614959	1.228816059	-1.190499659
407	0041-00368	71	1	67.86050168	1.826016132	1.535511232	0.328408319	1.382793578	0.088589896
408	0041-00383	81	1	3.269785058	2.630150353	1.770375238	0.064711866	-1.928725145	1.118632716
409	0041-00369	70	1	11.92425567	1.911879927	1.831617066	0.26150461	-0.515935397	0.218297503
410	0041-00367	70	2	43.62966812	1.055555556	0.514519205	0.419980576	0.900471245	-1.458486407
411	0041-00366	75	2	37.28637593	1.947411622	1.076431279	0.281460614	0.728919323	0.270276368
412	0041-00429	82	1	9.829136479	2.013615628	1.639781909	0.023481096	-0.726921329	0.364644001
413	0041-00424	86	2	85.93037474	2.551224774	1.571550174	0.289457758	1.640579147	1.032629932
414	0041-00422	79	2	15.02054202	2.871324853	0.992515365	0.303976573	-0.263870547	1.366281867
415	0041-00332	70	2	27.10222267	0.927150137	1.934146706	0.406748724	0.380581321	-1.824619983
416	0041-00410	74	2	35.58170973	2.010371397	1.176685605	0.45808233	0.677821228	0.360092428
417	0041-00416	70	1	83.71033855	2.590207448	1.433004909	-0.032849933	1.611998093	1.075435672
418	0041-00428	72	2	38.03674692	1.424734982	1.551226812	0.303276297	0.75067565	-0.611883842
419	0041-00423	84	1	8.454361387	2.406507056	1.922779098	0.413334548	-0.891440434	0.86778779

420	0041-00404	68	1	33.61773243	1.431347542	1.580203367	0.153734088	0.615823754	-0.598812932
421	0041-00430	93	1	5.912393658	3.102694481	0.927080808	0.381387139	-1.281948012	1.585039485
422	0041-00403	71	1	46.35682016	1.782822768	1.526568416	0.155745097	0.966675902	0.021016391
423	0041-00406	69	2	50.63688689	1.246605275	0.771763342	0.491739013	1.063105995	-0.98889852
424	0041-00426	68	2	42.65585225	1.074165163	1.666113638	0.251108172	0.875823293	-1.409154058
425	0041-00427	67	1	23.86014	2.908109182	1.704466998	0.09572896	0.241462809	1.402214544
426	0041-00419	85	2	2.370912829	4.070386632	1.629978623	0.031071063	-2.279724117	2.351329801
427	0041-00425	70	2	20.83290009	1.421760561	1.377672988	0.463610708	0.093314769	-0.617783114
428	0041-00402	76	1	47.62917029	1.461838005	0.374915587	0.609635663	0.996241967	-0.539313889
429	0041-00414	74	2	44.5298773	1.619864262	1.318749731	0.152826463	0.922771646	-0.249562282
430	0041-00401	73	2	16.31274739	2.135926133	1.677873016	0.164899249	-0.173755892	0.531098531
431	0041-00405	81	2	55.54879685	2.134740231	1.648651498	0.209478624	1.164198268	0.529530844
432	0041-00407	79	2	17.22559246	3.460937913	1.212527111	0.329586229	-0.114301181	1.893480395
433	0041-00408	72	2	3.680144173	2.419293194	1.744674657	0.329345035	-1.799629188	0.882745917
434	0041-00411	93	2	1.128101266	6.453554692	1.236174168	0.539193808	-3.090740782	3.652327093
435	0041-00412	74	2	44.01654502	1.528428794	0.565864833	0.383688504	0.910110999	-0.413571354
436	0041-00413	83	1	3.630365789	2.95747343	0.052348087	0.809798995	-1.81449961	1.449728129
437	0041-00418	80	2	33.52117048	1.580322625	1.643615951	0.184245211	0.612682843	-0.319322462
438	0041-00417	75	2	18.72081652	1.399825545	0.559950386	0.470920852	-0.023409339	-0.661672506
439	0041-00420	83	2	19.36421544	4.085011186	1.525319175	0.198745758	0.01348764	2.361453614
440	0041-00421	66	2	4.508433776	1.635595787	1.272324787	0.146073915	-1.577970378	-0.222280797
441	0041-00409	89	1	37.45572111	1.910300875	1.286999906	0.61119325	0.733867347	0.215965166
442	0041-00441	81	1	20.84120043	2.018048869	0.654025695	0.213675983	0.093749733	0.370851881
443	0041-00442	69	1	20.89022997	1.464214049	1.573518163	0.281101775	0.096315505	-0.534729528

444	0041-00443	79	2	10.90925371	1.827210884	1.31892273	0.304931679	-0.613076716	0.090436219
445	0041-00444	70	1	26.73749941	2.359669117	1.319943221	0.16560457	0.365787145	0.812306334
446	0041-00447	79	1	89.52675457	5.714541675	1.037795042	0.261788674	1.685348297	3.309029738
448	0041-00449	68	2	80.88285001	2.005844202	1.427120788	0.372851109	1.574478753	0.353728598
449	0041-00450	72	1	15.48736127	1.429663274	1.951952841	0.756981404	-0.230451522	-0.60213645
450	0041-00451	72	2	18.57126991	1.632202585	1.907856446	0.302297197	-0.03216695	-0.228143
451	0041-00453	76	2	43.70906623	1.33321052	1.189977429	0.155233761	0.902456549	-0.799304385
452	0041-00454	71	1	48.31981658	1.333338917	1.268841602	0.212774061	1.01196174	-0.799032546
453	0041-00455	73	1	39.00491487	2.330054257	1.640571529	0.124898732	0.778121166	0.776655113
454	0041-00456	73	2	23.91567315	1.842916365	1.684125277	0.124940555	0.244001255	0.114595218
455	0041-00457	67	2	4.563816745	1.852396884	1.591119334	0.367366034	-1.564638538	0.1290792
456	0041-00458	74	2	36.8943721	2.242424242	1.378682609	0.265505627	0.71737877	0.668446814
457	0041-00480	83	2	6.283041823	1.620644715	0.659328198	0.267734042	-1.215555089	-0.248202594
459	0041-00463	78	2	35.32361131	1.122419557	1.566960124	0.35071778	0.669871873	-1.285113144
460	0041-00464	83	2	6.23555775	2.284229442	0.798169951	0.209687817	-1.223838663	0.720586878
461	0041-00465	74	2	71.82268675	1.472318339	1.548065097	0.050155225	1.444756318	-0.519148811
462	0041-00466	71	1	6.183876937	2.076023392	0.794875306	0.358739725	-1.232926353	0.45080159
463	0041-00467	70	2	6.539012937	1.87332961	1.856261647	0.168001722	-1.171952275	0.160798645
464	0041-00468	72	1	83.17122239	2.075601513	0.791260838	0.317232243	1.604943061	0.450227902
465	0041-00469	82	1	25.03951518	1.936731048	0.815512197	0.272614198	0.294143795	0.254752259
466	0041-00471	97	2	23.37783536	3.26307619	1.919973084	0.317143979	0.219164664	1.727305754
467	0041-00472	68	2	13.24584095	1.314607432	1.398625916	0.287069315	-0.401164079	-0.838969639
468	0041-00473	73	2	28.72345611	1.786460926	1.368804296	0.513627214	0.444020441	0.026770881
470	0041-00476	78	1	9.325176174	3.154344811	1.122079536	0.118796083	-0.784392954	1.631643171

471	0041-00477	70	1	21.44223728	1.955866361	1.458236695	0.251993522	0.124794152	0.282504992
472	0041-00478	66	2	35.30315317	1.07755102	0.969657045	0.498909166	0.669239286	-1.400270434
473	0041-00479	71	2	60.44751965	0.955847024	1.331270098	0.291339177	1.256481019	-1.738575123
474	0041-00461	70	2	28.58514681	1.034097503	1.547813497	0.032437843	0.438749892	-1.516460981
475	0041-00462	69	2	15.44657428	2.141158496	1.781378485	-0.019611608	-0.233330976	0.538005006
476	0041-00481	79	1	41.04727651	1.306266628	1.456140427	0.014561489	0.83384964	-0.856936384
477	0041-00482	79	2	59.63739256	2.140553173	1.188832177	0.330648816	1.241747897	0.537206871
479	0041-00485	70	1	5.07423847	2.334093151	1.613284347	0.116031832	-1.44887555	0.781543847
480	0041-00486	76	2	53.18965302	1.194898825	1.775518647	0.33052602	1.116810864	-1.108478474
481	0041-00483	83	2	30.52702654	1.989387741	0.705370874	0.368118184	0.510516956	0.330474308
482	0041-00487	69	2	13.66116561	1.319803915	1.725827157	0.077331134	-0.367452406	-0.82783355
483	0041-00488	65	1	37.28541346	1.288514349	1.484684504	0.298719437	0.728891137	-0.895561195
484	0041-00489	66	2	13.19964471	1.595399188	1.135487756	0.120509412	-0.404978943	-0.29252033
485	0041-00452	70	1	12.90652124	1.4875	1.571538963	0.110089259	-0.429500567	-0.490191101
486	0041-00512	67	1	30.61995653	1.248822166	1.120562242	0.202869367	0.513835936	-0.983883118
487	0041-00513	66	1	58.02896667	1.147488025	1.1108092	0.172456974	1.211894122	-1.22276214
488	0041-00514	66	1	48.40168405	1.652250157	1.48600457	0.096500599	1.013810208	-0.193683392
489	0041-00515	67	2	49.60316533	1.373355204	1.763644837	0.059748162	1.040584296	-0.715561581
490	0041-00516	66	1	12.26045258	2.329711203	1.684860472	0.218075802	-0.485575219	0.776239485
491	0041-00517	71	2	23.84050042	1.869512136	1.695509391	0.137782877	0.24056366	0.155040521
492	0041-00518	86	1	17.99209325	1.848005402	1.088506502	0.367302088	-0.066762858	0.122379298
493	0041-00519	71	1	7.88002723	1.562845791	1.377315367	0.271728289	-0.968258666	-0.350713506
494	0041-00520	78	1	3.543853982	2.589905404	1.255081693	0.266703236	-1.840835333	1.075106488
495	0041-00521	79	2	33.21507915	4.801123291	0.896527718	0.193916127	0.602666349	2.817403513

496	0041-00522	74	1	49.20910098	1.251815833	0.549475819	0.413283902	1.031875019	-0.977124484
497	0041-00523	91	1	37.44267873	2.821193484	1.521335292	0.681342859	0.733487063	1.31656285
498	0041-00524	70	1	67.84714213	1.838687779	1.692045431	0.09754044	1.382578592	0.108110898
499	0041-00525	77	2	16.28627767	2.800462925	1.573366912	0.450432872	-0.175529135	1.29574409
500	0041-00526	68	2	36.8842575	2.796041235	1.831531559	0.264507989	0.717079377	1.29128365
503	0041-00530	70	1	26.47956069	1.717617051	1.211821835	0.447229827	0.355202104	-0.084160294
504	0041-00531	69	2	5.397594728	1.85384698	1.566841045	0.446543572	-1.381419623	0.131288065
505	0041-00532	77	2	7.746058266	5.235309822	1.422910776	0.234926343	-0.986982213	3.061788364
506	0041-00567	71	2	9.777852465	1.343995709	0.732200273	0.268073884	-0.732633426	-0.776561034
507	0041-00534	74	1	17.20761655	2.731162038	0.889080759	0.718942388	-0.115441265	1.225012249
508	0041-00535	72	2	9.31059877	1.782899588	0.99024348	0.128397882	-0.786101223	0.02113802
509	0041-00536	69	2	6.457944998	1.638227438	1.23572072	0.436347467	-1.185574139	-0.217742642
510	0041-00537	82	1	6.369035282	1.523434989	0.968977612	0.251126885	-1.200711676	-0.42280925
511	0041-00538	66	2	1.55529941	2.396310068	0.810094342	0.214374196	-2.740088017	0.855801557
512	0041-00445	70	1	67.58180693	1.6	0.804073655	0.182823926	1.378299935	-0.284391728
513	0041-00446	70	2	7.383683102	2.63085285	0.191814007	0.379543012	-1.039298085	1.119386561
514	0041-00539	78	1	49.15956882	1.969478044	1.829829799	0.204119983	1.030775371	0.302081804
515	0041-00540	82	1	32.3380068	2.095248327	0.868273049	0.420096558	0.573445578	0.476821492
516	0041-00541	72	2	9.346623954	2.285138612	1.691608065	0.294351144	-0.781884421	0.721710176
517	0041-00551	75	2	31.30180551	2.230671495	1.509713248	0.321235502	0.537884321	0.653613493
518	0041-00552	75	2	16.8043525	1.530856049	0.97065477	0.358912549	-0.141335409	-0.409092141
519	0041-00553	74	2	4.59955574	1.262286767	1.495569389	0.348435618	-1.556121028	-0.953611316
520	0041-00554	76	1	25.17688618	1.39148513	1.225421783	0.184934355	0.300117916	-0.678541413
521	0041-00490	82	1	43.17743232	3.173914015	0.662715886	0.101158029	0.889094001	1.649101251

522	0041-00555	72	2	5.336760076	2.410133149	1.401002017	0.14347857	−1.393796275	0.872037908
523	0041-00556	78	1	68.83252656	1.818527322	1.635256812	0.501595157	1.398323233	0.076989404
524	0041-00557	73	1	10.72133478	1.403991714	0.727277679	0.382041036	−0.632049741	−0.653283825
525	0041-00558	69	2	5.955898138	1.022654817	1.837793711	0.25971983	−1.27394283	−1.547870164
526	0041-00559	70	1	16.374902	1.207884931	1.030248857	0.147364545	−0.169603353	−1.077966195
527	0041-00560	77	2	48.15393038	2.936294357	0.774947262	0.009729068	1.00820661	1.429440935
528	0041-00561	70	2	14.27624266	1.935242919	1.214178709	0.082025563	−0.319364367	0.252582486
529	0041-00562	71	1	29.41854919	2.136365169	1.682631741	0.46779959	0.470129855	0.531678688
530	0041-00563	79	2	24.70157385	1.520643084	1.154613921	0.286735487	0.279306451	−0.42798712
531	0041-00564	68	2	15.96106018	1.379648572	1.468621251	0.329675489	−0.197554252	−0.70265583
532	0041-00565	72	1	36.91850655	1.336880367	1.392724625	0.182027291	0.718092821	−0.791544981
533	0041-00566	72	2	7.311657785	1.783919602	1.203061735	0.139768476	−1.050001749	0.022752494
534	0041-00529	75	1	11.94098602	1.438177087	1.567244124	0.126092545	−0.514404441	−0.585376352
536	0041-00570	69	1	12.09445023	1.413806049	0.864015856	0.251375278	−0.500460534	−0.633620407
537	0041-00571	66	2	46.52117563	2.052030986	1.07704019	0.157812365	0.97054042	0.417989079
538	0041-00572	72	2	17.16951905	1.891935228	1.082586132	0.150389836	−0.11786146	0.188695694
539	0041-00573	65	1	18.08060881	1.538030793	1.667650681	0.312183914	−0.061404085	−0.395893401
541	0041-00575	75	2	34.83783678	2.984810029	1.23475813	0.276906264	0.654751357	1.475699828
542	0041-00578	81	2	64.93587839	2.175464506	1.25721305	0.186965031	1.334689997	0.582873491
543	0041-00580	76	1	26.08401185	3.773972603	1.54205118	0.474916695	0.338767966	2.137900378
544	0041-00581	73	1	20.24237573	1.484011731	1.81248472	0.337552002	0.061916137	−0.496818436
545	0041-00582	69	1	13.07166654	2.291095458	1.416374389	0.576798743	−0.415617459	0.729058945
546	0041-00583	68	2	43.88470848	1.456099476	1.306261482	0.171437334	0.906835598	−0.550416658
547	0041-00584	70	2	6.292350205	1.826462953	1.11633096	0.360043184	−1.213938587	0.089280537

548	0041-00587	78	2	23.71297673	1.797256885	1.642313218	0.163191046	0.234707228	0.043778162
549	0041-00590	66	2	3.718685131	1.672101072	0.798812886	0.261610868	-1.788253258	-0.159971356
550	0041-00598	74	1	66.48504105	4.13986014	1.374986075	0.254610156	1.360434003	2.399102476
551	0041-00568	72	2	16.91537492	1.777868478	0.570389407	0.223262525	-0.134145032	0.013161259
552	0041-00586	66	1	66.60376532	1.331801741	1.822723941	0.616985669	1.362382149	-0.802288734
553	0041-00601	81	1	4.763735706	1.585266841	1.228281628	0.24989963	-1.517824481	-0.310504883
554	0041-00597	78	2	8.390237837	2.659530537	1.823498782	0.124439578	-0.899753907	1.149989801
555	0041-00592	70	1	47.65218801	0.944951789	0.677947659	0.200102376	0.996769535	-1.770935337
556	0041-00585	66	2	32.96377736	1.012883663	0.923774272	0.424804981	0.594373536	-1.574970587
557	0041-00600	70	1	19.29186167	1.143190267	1.678082847	-0.024590348	0.00940005	-1.233354299
558	0041-00603	66	2	15.83704438	0.989793814	1.518036972	0.005559566	-0.206071542	-1.640063767
559	0041-00604	66	2	47.33077075	1.131513403	1.285374139	0.058118518	0.989379463	-1.262335168
560	0041-00605	76	2	55.72451256	2.338506007	1.199674134	-0.004455265	1.167646873	0.78687557
561	0041-00606	67	2	49.95870821	0.967915942	1.675143577	0.053659703	1.048383032	-1.703156747
562	0041-00607	68	2	5.220222046	1.115146006	1.746046278	0.36893849	-1.41790471	-1.303464935
563	0041-00661	82	2	15.93131596	1.725811348	1.6986112	-0.014162357	-0.199591009	-0.070725603
564	0041-00662	76	2	17.4560409	1.663945024	0.717688976	0.047331733	-0.099789955	-0.173773761
565	0041-00663	75	1	36.11478895	2.380600484	1.202251651	0.23699332	0.694058957	0.83723526
567	0041-00665	70	2	63.65293481	1.357153483	1.241945751	0.221138973	1.312900749	-0.749060352
568	0041-00666	85	2	7.15776561	2.347176636	1.557685081	0.376686518	-1.073229393	0.797322398
569	0041-00667	69	2	14.41285326	1.126884422	1.803818432	0.132628966	-0.308965302	-1.273906723
570	0041-00668	69	2	11.10488592	1.679880603	0.854777473	0.370545774	-0.593669039	-0.146868719
571	0041-00669	70	2	6.486261364	1.206019471	1.158749965	0.051879375	-1.180796798	-1.082329064
573	0041-00671	79	2	17.72884543	1.648745657	1.045514101	0.225278415	-0.082857201	-0.199676987

574	0041-00672	71	2	6.856394766	1.314984062	0.811994445	0.081354319	-1.120199829	-0.838161041
575	0041-00674	75	2	22.37091963	2.140845066	1.248680454	0.217153665	0.171090973	0.537591768
576	0041-00675	81	1	16.36152401	3.35097408	0.836095815	0.118920489	-0.170495802	1.802337095
577	0041-00676	76	2	37.27726544	1.209868832	1.349683838	0.330585238	0.728652491	-1.073333707
578	0041-00681	83	1	24.27602775	2.563581822	1.213823754	0.525171069	0.260331375	1.046269246
579	0041-00683	68	2	16.99894406	1.443464644	1.571444046	0.082738289	-0.128763732	-0.575017258
580	0041-00684	71	2	38.03039786	1.559473281	1.385177625	0.408847183	0.750493371	-0.356811433
581	0041-00686	72	1	71.29048725	1.065199193	1.230421945	0.159406151	1.436635116	-1.432814414
582	0041-00688	65	2	6.751216633	1.118861856	1.580130869	0.192977938	-1.137079955	-1.294074622
583	0041-00719	70	1	63.89549198	1.928534869	1.853031583	0.027430829	1.317053759	0.242781025
584	0041-00720	75	2	19.03999938	2.578938703	0.829382044	0.048776468	-0.004949325	1.063128348
585	0041-00721	73	1	20.91822742	2.014392001	1.805470218	0.285227496	0.097777945	0.365732145
586	0041-00723	69	1	7.242461869	3.624461509	1.27966693	0.41144102	-1.060384711	2.023796892
587	0041-00724	65	2	24.28508815	1.716960118	1.32052488	0.304143988	0.260738833	-0.08524012
588	0041-00602	71	2		1.32450945	0.859886217	0.559243492		-0.817787329
589	0041-00588	70	1	25.89218487	1.470663901	1.385339684	0.234760207	0.330708048	-0.522322535
590	0041-00589	72	2	3.033531186	2.785430073	#NUM!	0.122055061	-2.010616264	1.280550654
591	0041-00728	75	1	23.78235785	0.951455446	1.413168699	0.167513432	0.2378974	-1.751574064
592	0041-00579	74	2	31.11336183	2.248618785	0.481948464	0.44489226	0.531290818	0.676233783
593	0041-00725	70	2	11.49327162	2.106275518	1.37625491	-0.021611544	-0.556132308	0.491638662
594	0041-00733	67	1	25.74802936	1.696599475	1.49294694	0.351915834	0.324611728	-0.118914168
595	0041-00734	66	2	19.13948909	1.583144344	1.060443671	0.32351518	0.000741465	-0.314286798
596	0041-00736	70	1	25.01588533	2.020725942	1.410743996	0.229579328	0.293112855	0.374593993
597	0041-00737	66	2	25.29812175	1.621906361	1.281930341	0.199520514	0.305363309	-0.246005963

598	0041-00738	75	1	8.248661421	1.18131062	1.398215877	0.305507417	-0.918336241	-1.140762545
599	0041-00739	73	2	27.60055134	1.413732394	1.403088278	0.210025777	0.400476232	-0.633767469
600	0041-00740	79	1	45.84743313	3.495462758	0.916383478	0.072364108	0.954610972	1.92149967
601	0041-00742	72	1	70.59930536	1.314151624	1.440917757	0.15036721	1.425996917	-0.839948536
602	0041-00743	67	2	2.631992601	1.67380809	1.661315026	0.543504679	-2.165654727	-0.157091106
603	0041-00744	66	2	8.15295765	1.247443095	1.848800428	0.118645476	-0.931079214	-0.987002023
604	0041-00748	67	2	17.62992556	1.420916128	0.420284664	0.223705663	-0.088966776	-0.619460155
605	0041-00762	80	1	49.39208484	2.440104725	0.911315186	0.096020743	1.035927814	0.906924475
607	0041-00764	68	1	22.88545017	1.44983921	1.246250479	0.152568444	0.195920797	-0.562578887
608	0041-00766	65	1	31.70021996	1.3	1.693657358	0.387408466	0.551694835	-0.870510829
609	0041-00767	66	1	56.4673826	1.325668303	1.35955946	0.161319841	1.182107302	-0.815318679
610	0041-00768	75	2	6.325929426	2.273771485	1.501062276	0.113943352	-1.208126997	0.707633609
611	0041-00770	72	2	6.297528826	2.346992721	1.751797658	0.122434872	-1.213040298	0.797101207
612	0041-00772	68	2	8.510490822	1.989361702	0.801124342	0.356746816	-0.884214972	0.330437362
613	0041-00773	71	1	55.94747172	1.536104743	0.799170164	0.370511743	1.17200706	-0.399430529
614	0041-00776	76	2	67.58702668	2.120586196	0.929954608	0.298713753	1.378384268	0.510752592
615	0041-00777	78	2	28.14295831	2.077886303	1.747780465	0.18642083	0.421726683	0.45333346
616	0041-00797	76	2	12.6423355	2.438502674	1.829863341	0.32645593	-0.452083319	0.905070574
617	0041-00779	70	2	29.0062303	2.078698548	1.449369747	0.31762178	0.454717577	0.454436666
618	0041-00780	87	1	5.032393632	3.328459082	1.101827311	0.387123236	-1.457917486	1.783307036
619	0041-00781	84	2	32.0918408	2.364058492	1.462491291	0.317791513	0.565101726	0.817552285
620	0041-00782	68	2	31.04670247	1.520526225	0.701774604	0.522243222	0.528948891	-0.428204054
621	0041-00783	74	2	68.79507419	2.639730559	1.506394629	0.373658218	1.397728945	1.128895867
622	0041-00785	71	2	3.842488661	1.857061992	1.49201548	0.181993915	-1.752492551	0.136179187

623	0041-00786	72	1	49.31924812	1.609968944	1.837557343	0.4215596	1.034316401	-0.266858742
624	0041-00787	72	2	7.614655695	1.810400275	0.584612595	0.268826401	-1.005664349	0.064346076
625	0041-00789	76	2	5.54050847	1.492299506	1.693016447	0.206817499	-1.352884472	-0.481097915
627	0041-00820	87	2	24.0575995	1.754032258	0.881650271	0.257774607	0.250462087	-0.024940206
628	0041-00802	72	2	4.009428736	1.520479173	0.743549623	0.173855995	-1.706054518	-0.428291404
629	0041-00803	73	2	3.117624301	2.09569378	1.381252291	0.244037576	-1.980758707	0.477421554
630	0041-00804	68	2	2.88903277	1.299684896	0.603082499	0.181980476	-2.063908384	-0.871195119
632	0041-00808	66	2	20.98519639	0.94036163	0.493823778	0.321327824	0.101268126	-1.784680542
633	0041-00809	67	2	14.11839307	1.456453521	0.460752468	0.113838072	-0.331504826	-0.549730395
634	0041-00810	71	2	6.242841813	1.950225657	1.321913038	-0.0267051	-1.222563874	0.274352368
635	0041-00812	71	2	13.92129061	1.650096426	1.149785269	0.16329663	-0.346856282	-0.197365317
636	0041-00815	79	2	15.33305673	2.416512397	0.79538233	0.290084866	-0.241385225	0.879499482
637	0041-00806	84	2	6.557880299	2.827204009	1.1436795	0.217509324	-1.168806218	1.322570346
638	0041-00826	89	2	9.828299881	1.661261942	1.185628742	0.383189027	-0.727014271	-0.17832911
639	0041-00823	70	2	23.19176807	1.870349921	0.816763485	0.451357148	0.210439105	0.156305208
640	0041-00825	78	2	25.76609969	1.95208378	0.992478399	0.220438116	0.325377788	0.277040552
641	0041-00833	72	1	18.67326276	2.583505192	1.365333859	0.271922866	-0.026186529	1.068122173
642	0073-00068	71	2	58.40647424	1.32930126	1.411048683	0.290498453	1.218974647	-0.807593524
643	0041-00835	72	2	15.32022333	1.795427482	1.271220208	0.412209339	-0.242299524	0.040903437
644	0041-00836	72	1	2.396918446	2.817740874	1.76646099	0.123623417	-2.267812408	1.31310619
645	0041-00837	69	1	42.27934064	1.732582094	1.185265096	0.254167869	0.866142365	-0.059672891
646	0041-00838	68	2	2.261968725	1.762531417	0.379653258	0.449901052	-2.331087791	-0.01129549
647	0041-00839	66	2	3.557029779	2.313402062	1.626128206	0.238693822	-1.836783155	0.756409163
648	0041-00840	66	2	9.984433766	1.627866229	0.354486596	0.246136867	-0.709804099	-0.235652388

649	0073-00061	67	1	39.07206727	1.094593716	0.551087501	0.364251118	0.779999453	-1.355974423
650	0073-00062	72	2	39.81044154	1.609178345	0.99932344	0.211618714	0.800441859	-0.268245247
651	0073-00063	84	1	61.62019136	1.097276677	1.59186639	0.039252951	1.277461357	-1.34906398
652	0073-00064	81	2	43.57264746	2.441799344	1.599996994	0.20660418	0.899043247	0.908884173
653	0073-00065	66	2	7.160550403	1.291814947	1.789723043	0.040316148	-1.072804652	-0.888339754
654	0073-00069	69	1	44.29019267	1.604312314	1.639213948	0.387709973	0.916878413	-0.276794031
655	0073-00070	73	1	32.44921071	1.840596106	0.854946406	0.111200305	0.577194053	0.111039064
656	0073-00071	68	2	34.15348153	0.964875445	1.64630757	0.205288917	0.633088043	-1.712037835
657	0073-00072	66	1	43.76858823	1.399553571	1.511204138	0.264958499	0.903942498	-0.662220999
658	0041-00884	76	2	7.914559035	2.150377134	1.533434981	-0.015528746	-0.963484086	0.550132215
659	0073-00074	66	2	21.17899525	1.299742196	1.641162539	0.145989527	0.11130582	-0.871070672
660	0073-00077	81	1	54.29224841	1.89968986	0.898426723	0.332514633	1.139214554	0.200241988
661	0073-00078	78	2	22.84984155	1.853873239	1.325905353	0.113857218	0.19422049	0.131328048
662	0073-00079	65	1	7.471141247	1.517647056	1.734737828	0.278682705	-1.026440445	-0.433554142
663	0073-00080	67	2	17.52335597	1.624988688	1.358883193	0.268080035	-0.095587298	-0.240646557
664	0041-00878	68	1	35.96671759	2.422663924	0.873386947	0.181170784	0.689572833	0.886676069
665	0041-00879	72	1	12.48941614	0.966251955	1.243617283	0.210850342	-0.465371592	-1.70801368
666	0041-00880	67	2	8.830366065	1.080900289	1.555900805	0.384293172	-0.843926312	-1.39151023
667	0041-00881	67	2	3.252768797	1.851252935	1.096542136	-0.014909615	-1.934422469	0.127335456
668	0041-00882	66	2	47.34035903	1.612395852	0.945978708	0.033785633	0.989600643	-0.262606825
669	0041-00885	71	2	17.33539992	1.242980436	0.512253195	0.26746576	-0.107362601	-0.99711845
670	0041-00886	74	1	18.52482416	1.509614264	1.675231547	0.207471672	-0.034901221	-0.448534555
671	0041-00887	66	2	21.41818623	1.401518653	1.238933865	0.094464293	0.123568687	-0.658260384
672	0041-00888	73	1	46.67912283	1.211399711	1.267754094	0.178865991	0.974241415	-1.069764233

673	0041-00889	65	2	7.360092851	2.903339348	1.330782691	0.146598882	-1.042792284	1.397580873
674	0041-00890	72	1	81.44322074	5.479059335	1.669122686	0.083287466	1.582017729	3.19024551
675	0041-00926	72	1	28.23430559	1.907300453	0.866883293	0.4628978	0.425265152	0.211528077
676	0041-00905	71	1	38.04860915	1.766169154	1.91085494	0.738706004	0.751016127	-0.005475493
677	0041-00906	66	2	55.43838254	1.374643498	1.450777111	0.280419112	1.162025686	-0.712914881
678	0041-00927	71	2	12.55184839	2.181090631	1.580338786	0.247032296	-0.459926852	0.590164247
679	0041-00897	73	1	32.62083996	1.648779378	1.743810551	0.138190082	0.582954214	-0.199619255
680	0041-00898	71	2	43.41473162	1.345865763	1.098707685	0.338673712	0.895078701	-0.772636115
681	0041-00900	80	2	38.57573929	1.897167556	1.51349514	0.217162547	0.766039982	0.196491573
682	0073-00073	72	2	29.63448738	1.970649157	1.637637121	0.129001746	0.478115549	0.303759815
683	0041-00903	81	1	5.900484202	2.682528834	1.586314258	0.278105689	-1.284149721	1.174294813
684	0041-00904	68	1	12.15887502	1.863749059	1.471797419	0.294609312	-0.494659489	0.146325418
685	0041-00596	71	2	16.18202948	1.818662815	0.770887652	0.428544399	-0.182541004	0.077199712
686	0041-00928	75	1	4.32356427	1.830556451	1.084893394	0.270387437	-1.62368901	0.095599907
689	0041-00933	71	2	6.012575073	1.964931602	1.209032988	0.259752187	-1.263601073	0.295558037
690	0041-00937	70	1	35.47231728	1.068000657	0.635841919	0.262583126	0.674459036	-1.425400287
691	0041-00938	67	2	2.237422849	0.744908087	0.779060512	0.293347438	-2.343001652	-2.442397374
692	0041-00941	66	2	21.14616299	1.045256399	1.54988956	0.02857152	0.109611772	-1.486163734
693	0041-00942	67	2	9.663268017	1.561223485	0.349748069	-0.127897311	-0.745505037	-0.353645198
694	0041-00943	77	1	19.8368167	1.033923495	1.325231575	0.019222835	0.039817096	-1.51693601
695	0041-00944	74	2	15.05135371	1.345672757	0.985124025	0.193465076	-0.261632967	-0.77304095
696	0041-00949	74	1	18.73280602	2.343096982	1.29747198	0.014488404	-0.022710252	0.792411832
697	0041-00947	80	1	33.88196979	2.08	1.177575562	0.12893946	0.624372808	0.456203423
698	0041-00950	69	1	57.79359731	1.049421441	1.272602836	0.369790265	1.207456188	-1.47493815

699	0041-00951	67	2	58.62004298	1.206155633	1.529968651	0.318063335	1.222960097	-1.082010384
700	0041-00958	74	1	12.05554666	1.50678733	1.761879728	0.020949933	-0.503978537	-0.453825494
701	0041-00895	72	2	9.730719786	2.137877855	1.768046133	0.081403349	-0.737909623	0.533676689
702	0041-00892	67	2	5.107642939	1.591447045	1.081186908	0.17805196	-1.441710792	-0.299521618
703	0041-00893	67	2	3.381534344	1.486596526	0.988144966	0.329982889	-1.892030617	-0.491906111
704	0041-00991	67	1	72.00523577	1.097918155	0.708220529	0.201792192	1.447528106	-1.347414243
705	0041-00967	69	2	19.74562087	1.47444565	0.529113803	0.172193114	0.034785605	-0.515073208
706	0041-00973	74	1	48.75997393	2.396173221	1.857364077	0.040569967	1.021863345	0.855640351
707	0041-00974	68	2	8.771218443	1.479484958	1.295470794	0.168628769	-0.851264869	-0.505442079
708	0041-00976	83	1	50.37619384	2.294663478	1.688063465	0.37951821	1.057469927	0.733451552
709	0041-00979	71	2	31.95036534	1.59726497	0.943059927	0.170110554	0.560277374	-0.289221091
710	0041-00980	72	2	7.757317877	2.025558886	1.702225351	0.360719004	-0.985396149	0.381337126
711	0041-00983	72	2	24.71381529	1.228800951	1.504475829	0.203376967	0.279847446	-1.02950475
712	0041-00985	69	2	15.24405453	1.465457951	0.889711588	0.306544873	-0.247741885	-0.5323325
713	0041-00986	80	2	8.99061306	3.477225111	1.392939796	0.089481539	-0.824288481	1.906733231
714	0041-00995	74	2	22.85973701	2.155629705	1.183100494	0.165973362	0.194693263	0.557018796
715	0041-00996	76	1	12.13267987	1.076878296	0.953789307	0.541232807	-0.49701448	-1.402033266
716	0041-00997	66	1	75.03111309	2.475409192	1.35907123	0.33357416	1.492476213	0.947472937
717	0041-00998	74	1	46.41703979	1.953172992	1.083956738	0.032166624	0.968093445	0.278615149
718	0041-00999	68	2	5.723779557	1.964824121	1.875241389	0.393646999	-1.317349824	0.295403627
719	0041-01000	72	2	28.6289621	2.293114687	1.66667744	0.29074071	0.440422315	0.731545667
720	0041-01024	68	1	53.00990797	1.877933771	0.757682899	0.293323681	1.113114642	0.167727785
721	0041-01025	67	1	20.8201243	1.792216624	1.456805604	0.360425776	0.092644938	0.0358508
722	0041-01026	79	1	6.709595866	2.159349352	1.72435705	0.273680272	-1.14383244	0.561885437

723	0041-01027	74	2	19.39887931	1.543259465	1.318483318	0.253390501	0.01544055	-0.386313404
724	0041-01028	70	2	26.2675463	1.76495517	0.826696362	0.334322911	0.346424166	-0.007416407
725	0041-01031	67	2	21.59903748	1.661428133	1.287776641	0.188438949	0.132750007	-0.178046736
726	0041-01032	65	2	40.59748861	1.423110876	1.419419507	0.246733679	0.821818486	-0.615103459
727	0041-01033	66	2	23.19060024	1.322921591	1.334434398	0.22048156	0.210384119	-0.82117338
728	0041-01034	69	2	36.36629487	1.12628866	1.608499169	0.153238738	0.701636855	-1.275399464
729	0041-01035	68	1	44.79202946	1.521977552	1.36531199	0.121534105	0.929181088	-0.42551103
730	0041-01037	71	2	3.134222043	1.537954763	1.560699056	0.051649711	-1.974960881	-0.396032943
733	0041-01042	71	2	10.29943491	1.588157895	1.65120074	0.182408247	-0.675886919	-0.305361672
734	0041-01043	77	2	1.872660923	2.472113848	0.496129761	0.186943561	-2.537325639	0.943712666
735	0041-01044	81	2	4.528222063	2.033316257	1.012813397	0.200893678	-1.573188212	0.39212698
736	0041-01045	79	2	14.62767216	2.521276596	0.272459148	0.393068467	-0.292810583	0.999298078
737	0041-01046	66	2	9.614809726	1.617948293	0.655927716	0.308204933	-0.750994486	-0.252903025
738	0041-01047	65	2	3.253851503	1.809581325	1.165175218	0.401620492	-1.934059074	0.063068882
739	0041-01048	69	1	47.00327536	2.210108565	0.982940694	0.208964638	0.981797844	0.627471723
740	0041-01054	72	2	7.870109455	2.004838438	0.512397729	0.257578106	-0.969633827	0.352312857
741	0041-01056	69	2	5.999512257	1.157033101	1.672128122	0.344413608	-1.265975955	-1.199378755
742	0041-01058	76	1	20.70858358	1.585850435	0.895980772	0.30207938	0.086779377	-0.309465909
743	0041-01059	71	2	5.059387298	1.428506644	0.778115945	0.063345784	-1.452076064	-0.604421064
744	0041-01066	65	2	7.300983489	1.663507109	1.316150395	0.200262226	-1.051597018	-0.174516754
745	0041-01061	70	2	30.96394578	1.217948718	0.704097926	0.154882265	0.526034414	-1.054544997
746	0041-01062	73	1	4.697610901	1.677117704	0.863381366	0.221024661	-1.533087555	-0.151515157
747	0041-01063	71	2	19.4586972	1.530286143	1.490856298	0.085629003	0.018802407	-0.410143197
748	0041-00994	70	1	49.83989714	2.492374423	0.671877042	0.224563543	1.045783135	0.966752846

749	0041-01064	66	1	41.65375306	3.121358671	1.28911376	0.184772646	0.849864913	1.601968954
750	0041-01067	69	2	6.806880948	2.128724603	1.697577137	0.396613286	-1.128113845	0.521565129
751	0041-01068	69	1	37.88341054	1.58303658	1.619654138	0.494343676	0.746264902	-0.31447895
752	0041-01069	66	2	23.05427054	0.955065037	0.832948155	0.32811948	0.203946109	-1.740885406
753	0041-01070	70	2	42.55040328	1.79286999	1.57844907	0.19949095	0.873120614	0.036879677
755	0041-01075	68	2	38.14981874	1.896414343	1.362751385	-0.019967053	0.753916804	0.195370653
756	0041-01076	68	2	16.09008891	1.366344319	1.628903681	0.253548798	-0.188762637	-0.730008559
757	0041-01077	68	2	37.32601254	1.376037397	1.581492479	0.277933231	0.73007946	-0.710054017
758	0041-01078	71	1	36.52190566	1.508597906	1.206558444	0.135560156	0.706299224	-0.450435647
759	0041-01080	79	1	83.86706255	1.880513115	1.572011598	0.138630237	1.614040507	0.171602208
760	0041-01081	73	1	8.014161377	1.244318182	1.56255343	0.178573501	-0.949828266	-0.9940821
761	0041-01082	76	1	10.02835652	2.031446541	1.923591432	0.274276367	-0.705011114	0.389530131
762	0041-01083	72	2	55.60634518	1.094601684	0.903858083	0.094931447	1.165328914	-1.355953874
763	0041-01095	71	1	21.0759706	1.133114233	1.001229765	0.307805398	0.105981211	-1.258344415
765	0041-01041	73	2	4.428779319	4.517326733	1.745124351	0.039256112	-1.597434864	2.645413214
766	0041-01102	84	2	9.058952756	1.779151725	1.323787584	0.054273695	-0.816019884	0.015197972
767	0041-01103	69	2	14.20717911	1.246469253	0.64628404	0.654881504	-0.324659551	-0.989206541
768	0041-01104	80	1	33.45579198	1.497920907	0.957077995	0.250212986	0.610551109	-0.470484673
769	0041-01105	65	2	1.7500201	1.578400925	1.152507856	0.09568157	-2.611285218	-0.322757093
770	0041-01106	73	2	50.62977106	1.656658849	1.524471315	0.175488882	1.06295254	-0.186161425
771	0041-01107	73	2	6.165080859	2.53033309	0.243043037	0.198217327	-1.236250351	1.009419393
772	0041-01108	72	2	59.80433547	1.925800172	1.704405964	0.219233085	1.244800257	0.23877544
773	0041-01125	71	2	16.25579104	2.546000839	0.789938777	0.403177695	-0.177575051	1.026844041
774	0041-01126	71	2	4.299991052	1.219849547	1.776732669	0.284611221	-1.629658773	-1.050142983

775	0041-01112	72	2	11.76739659	1.603233479	1.211008108	0.405858542	-0.530394592	-0.278692871
776	0041-01113	82	1	12.02443612	2.566471412	0.633467552	0.086306269	-0.506800005	1.049449198
777	0041-01114	70	1	20.95574671	1.509187549	1.070680391	0.204996773	0.099734689	-0.449332567
778	0041-01115	74	2	12.2698877	1.381619301	1.080064719	0.409336431	-0.484735242	-0.698626577
779	0041-01116	71	2	2.986560626	1.39911876	1.321303141	0.178743214	-2.027655688	-0.663098112
780	0041-01118	66	2	18.52579554	1.170241743	1.088840588	0.140388392	-0.034843966	-1.167336628
781	0041-01120	71	1	5.515369636	2.440500488	0.475171335	0.14585458	-1.357850122	0.907382267
782	0041-01121	71	2	31.23708735	1.051604847	1.267776867	0.068275586	0.535624367	-1.469071241
783	0041-01122	76	2	23.43585528	1.784037559	0.741574624	0.387478899	0.221871288	0.022939136
785	0041-01128	67	1	25.97312449	1.712873482	1.494670532	0.021852579	0.334116114	-0.091966778
786	0041-01129	66	1	62.16811418	2.211114594	1.369880808	0.251403993	1.287127787	0.628756341
787	0041-01130	73	1	26.08418949	1.509943695	1.414524197	0.233725286	0.338775403	-0.44791863
788	0041-01131	70	2	49.53357442	1.317089911	1.793567694	0.344611251	1.039051298	-0.833644192
789	0041-01133	67	2	32.56349084	0.895757961	1.416377347	0.178960753	0.581032861	-1.92185121
790	0041-01134	66	1	45.05709734	1.716446746	1.694899668	0.119615423	0.935623789	-0.086084257
791	0041-01135	69	2	12.2680228	1.873371689	1.512730955	-0.047809324	-0.484901216	0.160862049
792	0041-01158	74	2	7.605612208	1.049803516	1.65376321	0.234630334	-1.006961938	-1.473910619
793	0041-01137	66	1	30.44112811	1.643006736	1.088774575	0.272623953	0.507440108	-0.209519584
794	0041-01138	68	1	81.89298059	2.097830793	0.881134178	0.021108023	1.588031166	0.480298517
795	0041-01142	74	2	36.36509219	1.878365191	1.483460743	0.215639344	0.701600743	0.16837619
796	0041-01147	71	1	32.34556156	2.428525641	1.913246678	0.321770456	0.573700642	0.893497618
797	0041-01148	71	2	4.232897963	1.899214631	1.560684693	0.273780032	-1.646830506	0.199535751
798	0041-01159	65	1	13.7238805	1.679278142	1.509814695	0.385342693	-0.362451123	-0.14788124
799	0041-01161	71	1	8.342032039	2.459337349	0.6266378	0.278574047	-0.906045622	0.929086036

800	0041-01163	69	2	71.66910114	2.219784877	1.137476928	0.225122635	1.442418842	0.639803436
801	0041-01164	65	2	34.3772761	1.912514514	0.921271854	0.390818105	0.640219677	0.219234277
802	0041-01165	65	2	70.07424677	0.964657432	1.855331958	0.346310888	1.417845731	-1.712675712
803	0041-01167	70	2	8.18152274	1.823304086	1.536271462	0.28160474	-0.927260174	0.084394323
804	0041-01109	72	2	22.60895029	1.234103534	1.845558438	-0.015626886	0.182647897	-1.017349981
805	0041-01174	80	2	36.05608783	1.82427032	0.912834142	0.260859105	0.692282691	0.085889817
806	0041-01175	68	2	13.55258453	1.571650556	1.354280399	0.091351596	-0.376165885	-0.334855181
807	0041-01178	67	1	23.14289586	1.470575341	1.556978603	0.261089192	0.20813565	-0.52249252
808	0041-01179	66	2	40.1690787	1.220099562	1.132022125	0.196355991	0.810234569	-1.049564498
809	0041-01182	65	1	69.15357551	1.043498389	1.364417701	0.167487279	1.403404362	-1.490915336
810	0041-01183	69	1	6.234968562	1.471818952	1.603891871	0.086395271	-1.223941842	-0.520106414
811	0041-01184	65	2	18.83511525	1.438053097	1.83981464	0.018491783	-0.01676292	-0.585619721
812	0041-01185	70	2	39.73609791	1.455808079	0.794834268	0.167854391	0.798400845	-0.550981613
813	0041-01188	67	2	39.30207056	1.320411401	1.274968282	0.157774922	0.78640839	-0.826534569
814	0041-01189	71	1	29.70844025	2.232035928	1.599185217	0.163104125	0.480837058	0.65533957
815	0041-01190	65	2	44.05157109	1.20342104	1.594415431	0.120709265	0.910979552	-1.088417437
816	0041-01191	65	2	54.21312618	0.673224843	1.472879851	0.348701181	1.137622085	-2.728009856
817	0041-01194	68	1	35.1798573	0.841818866	1.643961402	0.0804176	0.665419069	-2.097160678
818	0041-01195	81	1	46.57482403	2.049729929	1.734104451	-0.171839866	0.971798908	0.414821968
819	0041-01196	77	2	20.63016991	3.229865772	1.546294074	-0.074781345	0.082636917	1.698429372
820	0041-01186	66	2	33.20129932	1.187447028	1.668151223	0.311696642	0.602213251	-1.126137381
