# Supplementary material for: Joint Dietary and Gut Microbial Profiling and the Fatty Liver Index in Community-Dwelling Older Japanese: A Cross-Sectional, Hypothesis-Generating Analysis from the Kyotango Longevity Study
Source: Nutrients. 2026 Jul 14;18(14):2300. doi: 10.3390/nu18142300 (PMC13415844; doi:10.3390/nu18142300)
Supplement: Supplementary file 1 [file nutrients-18-02300-s001.zip › Supplementary Table S3.pdf]

Supplementary Table S3. Cluster derivation diagnostics

(A) PCA eigenvalues — Food groups (n = 31)

Component	1	2	3	4	5	6	7	8	9	10
Eigenvalue	4.093	2.442	2.024	1.771	1.521	1.41	1.249	1.228	1.125	1.059
Cumulative variance (%)	18.1	28.8	37.7	45.6	52.3	58.5	64	69.4	74.4	79

(B) PCA eigenvalues — Microbiome genera (n = 47)

Component	1	2	3	4	5	6	7	8	9	10
Eigenvalue	8.746	2.872	2.696	2.306	1.73	1.507	1.476	1.332	1.27	1.225
Cumulative variance (%)	28.8	38.3	47.2	54.8	60.5	65.4	70.3	74.7	78.9	82.9

(C) Cluster diagnostics across k = 2–6

Modality	Metric	k=2	k=3	k=4	k=5	k=6
Food	Silhouette	0.282	0.204	0.201	0.183	0.171
Food	Calinski-Harabasz	342.5	241.6	220.2	188.3	174.5
Microbe	Silhouette	0.303	0.205	0.203	0.222	0.228
Microbe	Calinski-Harabasz	394.4	290.4	247.6	234.1	221.2

(D) Bootstrap Jaccard stability at k = 4 (B = 100, mean ± SD)

Modality	Cluster 1	Cluster 2	Cluster 3	Cluster 4	Mean	Min
Food	0.759 ± 0.127	0.662 ± 0.199	0.784 ± 0.117	0.570 ± 0.234	0.694	0.570
Microbe	0.573 ± 0.172	0.748 ± 0.109	0.495 ± 0.215	0.608 ± 0.206	0.606	0.495

PCA: Varimax-rotated, applied separately to 31 energy-adjusted food group z-scores and 47 CLR_Z-transformed genera. Component
