# Supplementary material for: Joint Dietary and Gut Microbial Profiling and the Fatty Liver Index in Community-Dwelling Older Japanese: A Cross-Sectional, Hypothesis-Generating Analysis from the Kyotango Longevity Study
Source: Nutrients. 2026 Jul 14;18(14):2300. doi: 10.3390/nu18142300 (PMC13415844; doi:10.3390/nu18142300)
Supplement: Supplementary file 1 [file nutrients-18-02300-s001.zip › Suuplementary Table S1.pdf]

Supplementary Table S1. Aggregation of BDHQ items into 31 food groups

Food group	BDHQ items	N items
Dairy	Low-fat milk; Milk	2
Red_meat	Pork/Beef; Ham; Liver	3
Poultry	Chicken	1
Fish_oily	Fatty fish; Canned tuna	2
Fish_lean	Lean fish; Fish with bone; Dried fish	3
Fish_dishes	Raw fish; Grilled fish; Boiled fish	3
Seafood_other	Squid/Octopus/Shrimp/Shellfish	1
Eggs	Eggs	1
Soy	Tofu/Fried tofu; Natto	2
Potato	Potato	1
Pickles	Pickles(Green leafy); Pickles(Other)	2
Green_yellow_veg	Green leafy vegetable; Carrots/Pumpkins; Tomatoes	3
Light_veg	Fresh lettuce, Cabbage); Cabbage; Radishes/Turnips; Root vegetables	4
Mushrooms	Mushrooms	1
Seaweed	Seaweed	1
Western_sweets	Western sweets; Ice cream	2
Japanese_sweets	Japanese sweets; Rice crackers	2
Fruits	Citrus fruits; Persimmons/Strawberries; Other fruits	3
Mayonnaise	Mayonnaise	1
Bread	Bread	1
Noodles	Soba noodles; Udon noodles; Ramen; Pasta	4
Green_tea	Green tea	1
Other_tea_coffee	Black tea/Oolong tea; Coffee	2
Soft_drinks	Cola; 100% juice	2
Sugar_added	Sugar; Cooking sugar	2
Rice	Rice	1
Miso_soup	Miso soup	1
Alcohol	Sake; Beer; Shochu; Whiskey; Wine	5
Fried_foods	Tempura/Fried fish; Fried food; Stir-fried food; Cooking oil	4
Meat_dishes	Grilled meat; Hamburg steak; Simmered food	3
Salt_soy	Noodle soup; Soy sauce; Cooking salt	3
