# Supplementary figures and images for: Joint Dietary and Gut Microbial Profiling and the Fatty Liver Index in Community-Dwelling Older Japanese: A Cross-Sectional, Hypothesis-Generating Analysis from the Kyotango Longevity Study
Source: Nutrients. 2026 Jul 14;18(14):2300. doi: 10.3390/nu18142300 (PMC13415844; doi:10.3390/nu18142300)

**Supplementary Figure S1. Age-residualization of Log[FIB-4] Z (n = 701)**

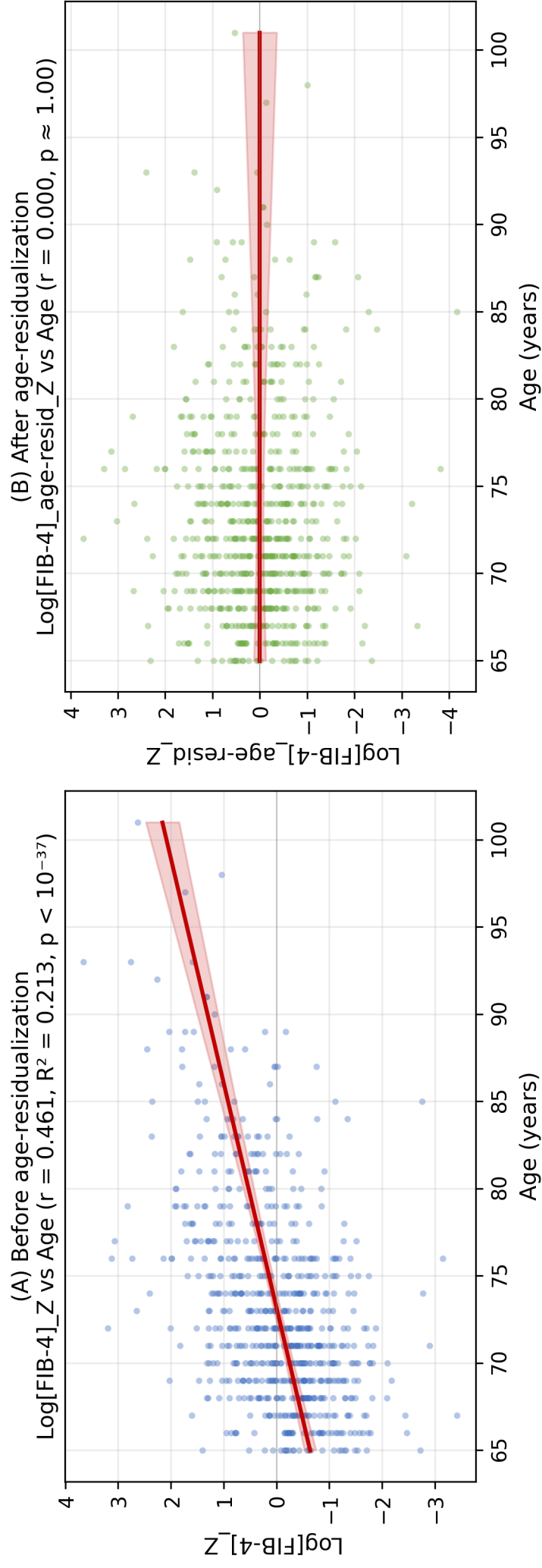

Supplement: Supplementary file 1 [file nutrients-18-02300-s001.zip › Figure S1_FIB4_age_adjustment.pdf]
